# Artesunate-mycophenolate Mofetil Dimer Micelles Alleviate Allogeneic Skin Graft Rejection by Inhibiting the TLR-4 Pathway in Macrophages

**DOI:** 10.7150/thno.108173

**Published:** 2025-06-12

**Authors:** Wentao Zhao, Zhentao Yang, Hong Tang, Jintao Zheng, Zhi Liang, Ruiqi Sun, Ning Wang, Rong Su, Hangxiang Wang, Yiting Qiao, Shusen Zheng, Penghong Song, Haiyang Xie

**Affiliations:** 1 Division of Hepatobiliary and Pancreatic Surgery, Department of Surgery, The First Affiliated Hospital, Zhejiang University School of Medicine, Hangzhou 310003, China.; 2 NHC Key Laboratory of Combined Multi-organ Transplantation, Hangzhou 310003, China.; 3 Key Laboratory of the Diagnosis and Treatment of Organ Transplantation, Research Unit of Collaborative Diagnosis and Treatment for Hepatobiliary and Pancreatic Cancer, Chinese Academy of Medical Sciences (2019RU019), Hangzhou 310003, China.; 4 Key Laboratory of Organ Transplantation, Research Center for Diagnosis and Treatment of Hepatobiliary Diseases, Zhejiang Province, Hangzhou 310003, China.; 5 State Key Laboratory for Diagnosis and Treatment of Infectious Diseases, Zhejiang Province, Hangzhou 310003, China.; 6 The Fourth School of Clinical Medicine, Zhejiang Chinese Medical University, Hangzhou 310053, China.

**Keywords:** Artesunate, Mycophenolate mofetil, Skin allograft, T cell, Macrophage

## Abstract

**Background:** Organ transplantation continues to be an essential therapeutic option for patients afflicted with end-stage organ failure. However, long-term administration of immunosuppressive agents has the potential to trigger severe adverse effects, including concurrent myelosuppression and systemic toxicity. Targeted delivery of small molecule compounds to immune organs, combined with chemical modification, may well offer a solution to these unmet needs.

**Methods:** Overall, we carried out molecular editing on artesunate (ART) and mycophenolate mofetil (MMF). These compounds were then further optimized through PEGylation using amphiphilic polymers. The PEGylated ART-MMF nano-prodrugs (AMNPs) is capable of self-assembling to generate immunosuppressant nanoparticles, enabling targeted therapeutic delivery to immune organs. In addition, leveraging the allogeneic skin transplantation mouse model empowers us to comprehensively assess the immunotherapeutic efficacy of AMNPs.

**Results:** AMNPs exhibit a more potent immunosuppressive effect and enhanced biocompatibility.* In vivo*, AMNPs more effectively suppressed the expression of Tumour Necrosis Factor-α (TNF-α) and interleukin 6 (IL-6) in macrophages and proliferation of CD45.1^+^ C57BL/6 mice T cells in CD45.2^+^ C57BL/6 mice.* In vitro*, AMNPs effectively inhibited the expression of histocompatibility complex II (MHC-II) on Lipopolysaccharide (LPS) induced macrophages and further promoted the expression of CD206 on macrophages induced by tumor supernatants. After depleting macrophages in C57BL/6 mice, the significant effect of AMNPs on T cell anti-inflammatory differentiation was abolished.

**Conclusion:** These findings suggest that targeted delivery of AMNPs using a prodrug-assembled nanoparticles may provide a therapeutic option for combating organ rejection.

## Introduction

Solid organ transplantation remains the primary treatment option for patients with end-stage organ disease 1. Enhancing transplant success rates, reducing rejection responses, and improving long-term outcomes are important areas that require further exploration. Following transplantation, the administration of immunosuppressants (ISAs) can effectively manage acute rejection reactions and enhance the survival rate of the graft. However, the severe systemic toxicity associated with ISAs significantly reduces the survival rate of long-term recipients, closely linked to serious complications related to high doses of immunosuppressants, such as opportunistic infections, chronic kidney disease, cardiovascular diseases, diabetes, and malignancies 2. Although attempts have been made to address these issues by reducing ISA dosages, the outcomes have been inconsistent, leading to an increased risk of graft rejection and subsequent chronic organ failure 3. Therefore, developing novel immunosuppressants with immune organ-targeting effects to reduce drug toxicity and enhance the therapeutic efficacy of immunosuppression is an important direction for current research.

Artesunate (ART) is a natural small molecule compound that shows promising potential in post-transplant immunotherapy 4. Previous research has indicated that ART regulates inflammasome activity by inhibiting the Toll-like receptor-4 (TLR-4) signaling pathway, suppresses inflammation-related signaling pathways, and promotes the polarization of macrophages to the M2 phenotype 5,6. Mycophenolate mofetil (MMF) acts on lymphocyte proliferation, reducing T cell proliferation and activation, and modulating the Type 1 CD4^+^ T cells (Th1)/Type 2 CD4^+^ T cells (Th2) balance. It is used clinically to support tacrolimus (TAC) in enhancing immunosuppressive efficacy 7. However, long-term use of MMF in clinical settings often results in considerable side effects, making it difficult for patients to tolerate extended treatment 8. As a natural small-molecule drug, artesunate can inhibit immune responses and has low toxicity and side effects 9. Therefore, the combined use of ART and MMF post-transplantation is likely to yield more favorable results and enhanced safety, thereby decreasing the risks linked to the long-term administration of high-dose immunosuppressive medications. In recent years, nanoparticle drug delivery system demonstrates great potential in enhancing the efficacy of encapsulated drugs while reducing drug-related toxicity 10. Specifically, nanoparticle therapeutics can be designed to preferentially accumulate in various pathological sites, such as critical immune organs, inflamed areas, and antigen sampling sites 11. Once accumulated in the target tissue, the nanoparticle carriers can act as a local drug reservoir, continuously releasing therapeutically active compounds 12. Our therapeutic strategy involved molecular editing of MMF and ART to create a novel ART-MMF compound through an esterification reaction, which can then self-assemble into ART-MMF nanoparticles (AMNPs). The treatment regimen involving AMNPs enhanced the pharmacokinetic characteristics of the drug, leading to a remarkable increase in the animals' tolerance to the medication. It promoted the accumulation of the drug within the spleens, thereby enabling effective immunosuppressive regulation of cytotoxic CD8^+^ T cells.

Macrophages are typically regarded as scavengers *in vivo*, and this characteristic represents a disadvantage in cancer treatment using nanodrugs. However, our previous research in a cardiac transplantation model has shown that macrophages more effectively promote M2 polarization and enhance immunosuppressive effects after phagocytosing nanoparticles 13. Interestingly, the nanoparticles we designed preferentially accumulate in the spleens and are mainly phagocytosed by macrophages, further influencing the Th1/Th2 balance of T cells by regulating macrophage differentiation. The immunomodulatory effects of AMNPs are significantly diminished after macrophage depletion. Overall, we propose a simple and cost-effective method to construct a nanoparticle therapeutic platform capable of efficiently delivering a combination of the natural and traditional drug. Compared to conventional treatments, our approach highlights the superior immunosuppressive effects, extends graft survival, and mitigates the adverse reactions associated with traditional immunosuppressive drugs, providing a novel therapeutic strategy for combating allograft rejection.

## Results

### Preparation, characterization, and distribution of self-assembled AMNPs prodrug

**Figure 1:**
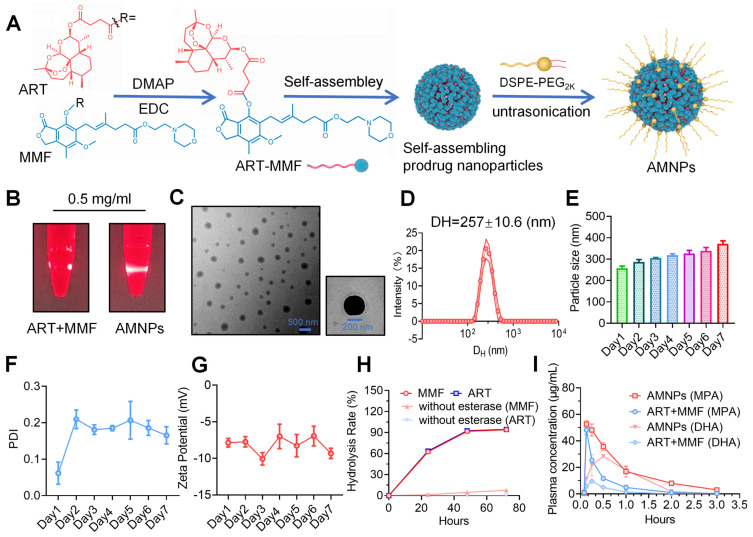
** Characterization of prodrug-based self-assembled nanoparticles. (A)** Illustration of the reaction process between ART and MMF for prodrug synthesis. **(B)** Photographs of AMNPs (10 μM) in water under bright light and laser irradiation. **(C)** Representative TEM images and corresponding nanoparticle diameters of AMNPs (n = 3), analyzed using ImageJ software. Scale bars: 200 nm and 500 nm. **(D-G)** Representative distribution of D_H_, PDI, and zeta potential of AMNPs in double-distilled water (n = 3). **(H)** Hydrolysis rate of ART-MMF self-assembled nanoparticles prodrug with or without esterase at 60 U/mL (n = 3). **(I)** The pharmacokinetic evaluation of AMNPs and the combination of ART and MMF in SD rats was conducted at 0.083, 0.166, 0.25, 0.5, 1, 2, and 3 h after drug administration (n = 3). Data are represented as mean ± SD, *p < 0.05, **p < 0.01, ***p < 0.001, ****p < 0.0001, and ns = not significant, p > 0.05.

Our previous research utilized PUFAylation technology to modify the MMF molecule by covalently conjugating it to various unsaturated fatty acids. This strategy facilitates the creation of stable nanoparticles designed to extend circulation time in the bloodstream after systemic administration 14. Clinically, MMF is usually combined with Tacrolimus, a hydrophobic macrolide with potent immunosuppressive activity, to treat and prevent graft rejection. However, the application of MMF and TAC is often associated with adverse effects, such as leukopenia and an elevated risk of tumor recurrence 15. In contrast, the combination of immunosuppressants and small natural molecules can reduce toxicity. Transforming them into nanoparticles may enhance bioavailability and improve the efficacy of immunotherapy. Based on our previous screening of artemisinin derivatives using carboxyfluorescein succinimidyl ester (CFSE) proliferation assays 16, we conjugated the carboxyl group of ART with the hydroxyl group of MMF through esterification, producing the ART-MMF self-assembled nanoparticle prodrug and which were characterized with ^1^H nuclear magnetic resonance (NMR) and mass spectrum (MS) (Figure S1A-B). The molecular weight of AMNP-Na^+^ was determined to be 822.367 Da, which matches the theoretical value (approximately 822.91 Da), thereby confirming the identity of the compound as AMNP-Na^+^. This production approach was expected to impart amphiphilicity to the prodrugs, allowing them to self-assemble into nanoparticles in aqueous media 17. To obtain better stability and prolong the circulation of nanoparticles *in vivo*, 1,2-distearoyl-sn-glycero-3-phosphoethanolamine-N-[methoxy (polyethylene glycol) 2000] (DSPE-PEG_2k_) was used for surface PEGylation of ART-MMF nanoparticles (Figure 1A). To prepare PEGylated ART-MMF nanoassemblies named AMNPs, a solution of ART-MMF prodrug and DSPE-PEG_2k_ at 10:1 w/w ratio was dissolved in acetone was injected into deionized water, and then acetone was completely removed by rotary evaporation method. AMNPs exhibited the Tyndall effect in deionized water (Figure 1B), revealed the formation of uniformly spherical nanostructures via Transmission electron microscopy (TEM) (Figure 1C), and showed a stabled monomodal distribution and low polydispersity index (PDI, < 0.2) with a hydrodynamic diameter (D_H_) of 257 ± 10.6 nm within 24 h (Figure 1D-F). Moreover, a negative surface charge with a zeta potential between -5.8 mV and -10.9 mV was observed within 7 days in double distilled water (Figure 1G). Furthermore, we conducted additional tests on the particle size and stability of the nanoparticles in 5% serum, we found that the drug could still maintain a relatively stable and favorable state and keep its nanoscale particle size within 7 days in 5% serum (Figure S1C-D). Moreover, we confirmed that the AMNPs prodrug could be hydrolyzed *in vitro* by 60 U/mL of esterase, resulting in the release of ART and MMF over a 72-hour period (Figure 1H). Interestingly, AMNPs could be phagocytosed by Bone marrow-derived macrophages (BMDMs) *in vitro*, and the efficiency of BMDM in phagocytosing AMNP is concentration-dependent (Figure S2A-C). Furthermore, AMNPs effectively reduce the average fluorescence intensity of reactive oxygen species (ROS) in macrophages (Figure S2D). Quantitative determination of the critical micelle concentration (CMC) revealed that AMNPs exhibited a CMC value of 0.0118 mg/mL (Figure S1E). This substantially lower value compared to the therapeutic dosage confirms the maintenance of stable nanostructures during *in vivo* administration, effectively circumventing premature micelle dissociation.

**Figure 2:**
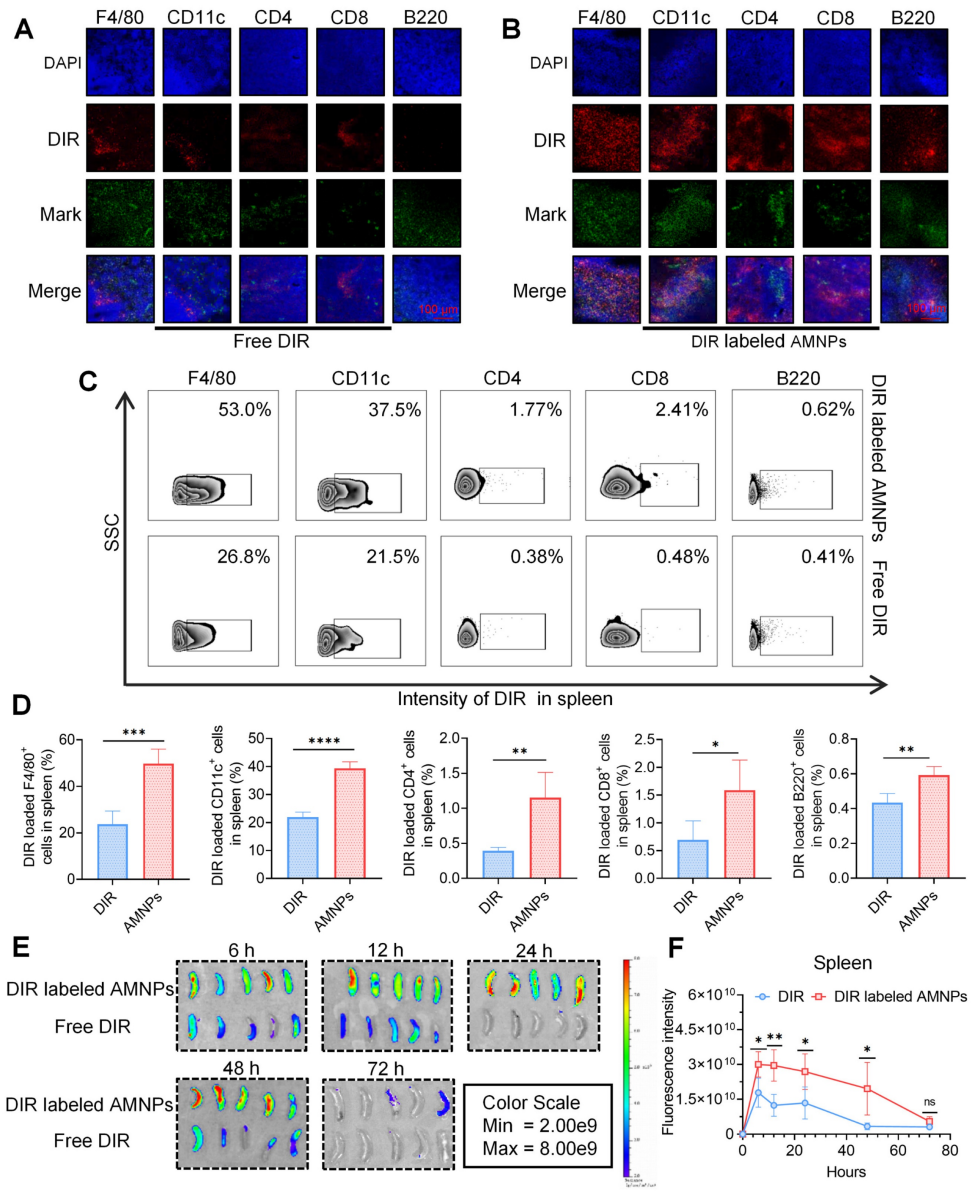
** Biodistribution of AMNPs in normal C57BL/6 mice. (A)** Immunofluorescence staining for immune cells in mouse splenic samples with free DIR. Scale bar: 100 μm (n = 3). **(B)** The immunofluorescence staining for immune cells in mouse splenic samples with DIR labeled AMNPs. Scale bar: 100 μm (n = 3). **(C, D)** The representative flow cytometry diagram and flow cytometry analysis of immune cells in mouse splenic samples with free DIR or DIR labeled AMNPs (n = 3). **(E)** The fluorescence images for the evaluation of free DIR or DIR labeled AMNPs distribution in spleens in 6, 12, 24, 48, and 72 h (n = 5). **(F)** Quantitative analysis of DIR or DIR labeled AMNP accumulation in the spleens (n = 5). Data are represented as mean ± SD, *p < 0.05, **p < 0.01, ***p < 0.001, ****p < 0.0001, and ns = not significant, p > 0.05.

Compared to MMF, ART is rapidly metabolized in the body 18. Next, we detect the metabolite Dihydroartemisinin (DHA) of ART, and mycophenolic acid (MPA) of MMF in the peripheral blood of SD rats, and the results showed that AMNPs treatment enhances blood drug concentration, half-lives, and bioavailability of DHA and MPA. (Figure 1I, Figure S2E). Specifically, these drugs in the AMNP group exhibit significantly higher peak concentrations (C_max_) and a substantially expanded area under the plasma concentration-time curve (AUC). This parametric characteristic underscores a notable prolongation of drug action duration *in vivo* and a marked increase in drug exposure levels, thereby providing a robust foundation for maintaining a stable and effective therapeutic concentration window. Notably, the drug clearance rate (CL) of the AMNP group shows a significant downward trend (Table S3). This characteristic enables the drug to act on the target sites continuously for a longer period and maintain a long-term binding with the target sites. Spleen is the crucial immune effector organ that coordinates both innate and adaptive immune responses, balancing pro-inflammatory and anti-inflammatory regulation. And the modulation of splenic immune activity is a key target in organ transplantation therapy 19. Through analyzing the drug concentrations in the major organs and allografts, we found that MPA mainly accumulates in the livers, spleens and allograft. Moreover, the drug concentrations within the organs in the AMNPs group are higher than those in the free drug group (Figure S2F). However, DHA could mainly be detected in the hearts and the allografts, and the drug concentration of DHA in the allografts of the AMNPs group was higher than that in the free drug group (Figure S2G). Next, we intraperitoneally injected 1,1'-dioctadecyl-3,3,3',3'-tetramethylindole tricarbocyanine iodine (DIR) labeled AMNPs into C57BL/6 mice and tracked their distribution and used immunofluorescence and flow cytometry to investigate the immune cells in the spleens that are primarily responsible for the phagocytosis of the nanoparticles. We found that macrophages are the primary immune cells that engulf AMNPs (Figure 2A-D), and AMNPs mainly accumulate in the livers and spleens, where they exert a sustained-release effect in the spleens and lymph nodes within 48 and 72 h, respectively (Figure 2E-F, Figure S3A-D). These results demonstrate that AMNPs were capable of targeting macrophages within the spleens, thereby exerting their immunomodulatory functions.

### AMNPs enhanced anti-rejection effects and prolonged the allograft survival

**Figure 3:**
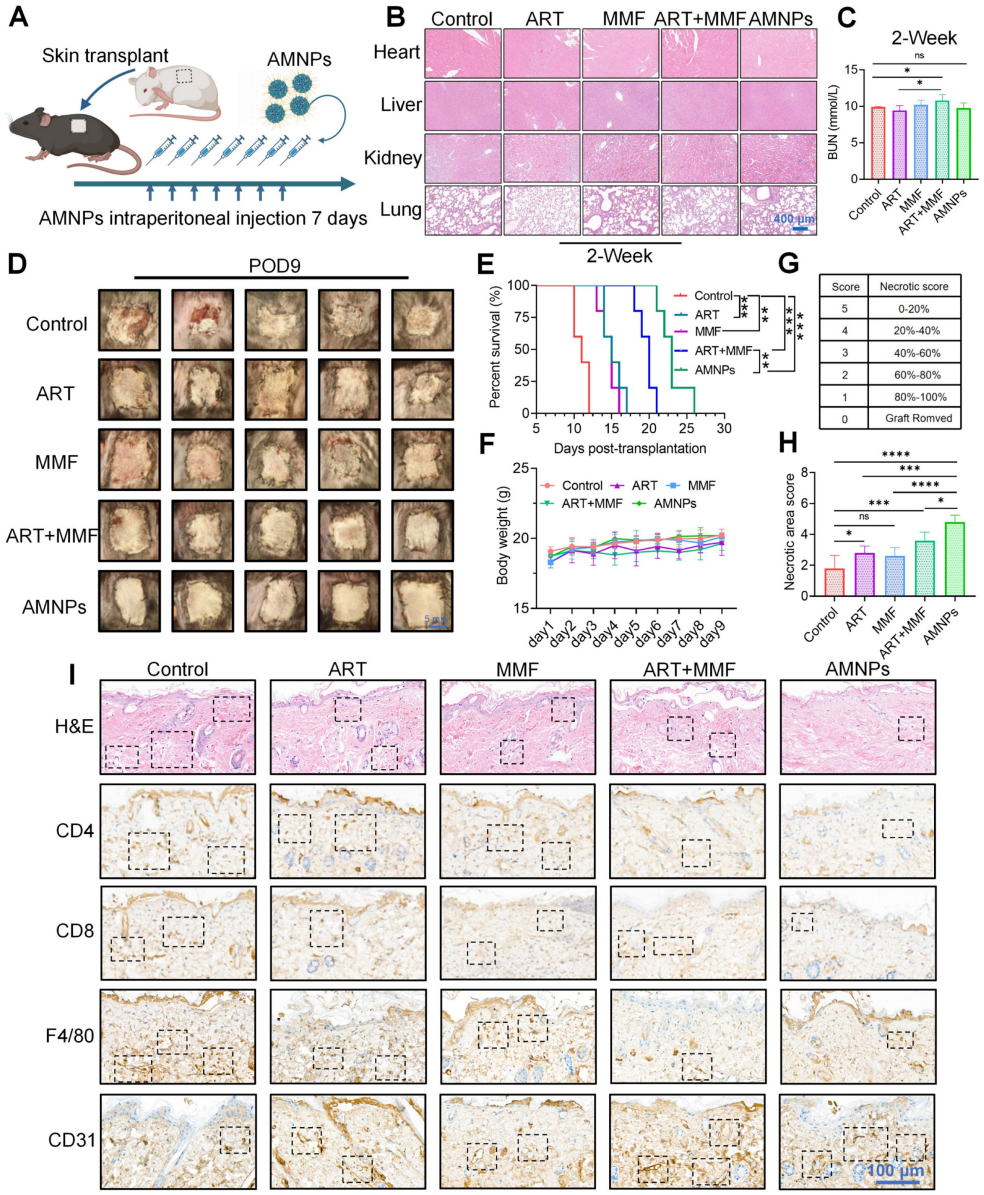
** The effects of ART (17.7 mg/kg), MMF (19.9 mg/kg), the combination of artesunate (17.7 mg/kg) and mycophenolate mofetil (19.9 mg/kg), and AMNPs (ART: 17.7 mg/kg; MMF: 19.9 mg/kg) on the allogeneic skin transplantation model after 7-day treatment. (A)** Shematic of the skin transplantation procedure and the drug administration protocol. **(B)** Representative images of H&E staining of the murine major organs at 14 days post-administration, and no toxic effects on these major organs were found. Scale bar: 400 μm (n = 5). **(C)** The concentration of BUN in plasma at 14 days post-administration. Scale bar: 5 mm (n = 5). **(D)** The appearance of mouse skin allografts treated with different drugs on POD9 (n = 5). **(E, F)** The graft survival analysis in the skin transplantation treatment model and the effects on body weight (n = 5). **(G, H)** Different degrees and statistical analysis of graft rejection on POD7, the lower the rejection, the higher the necrosis score (n = 5). **(I)** The representative photographs of H&E and immunohistochemical staining for CD4, CD8, F4/80, and CD31 on skin grafts on POD7, cell populations with significant differences are indicated by boxes. Scale bar: 100 μm (n = 3). Data are represented as mean ± SD, *p < 0.05, **p < 0.01, ***p < 0.001, ****p < 0.0001, and ns = not significant, p > 0.05.

We systematically evaluated the immunotherapeutic efficacy of AMNPs treatment in the allogeneic skin graft mice model and carried out the administration regimen in a BALB/c to C57BL/6 murine skin transplantation model (Figure 3A). Since drug treatment in clinical practice often leads to subclinical damage, we first evaluated the safety of the AMNPs treatment regimen in mice. Through pathological staining, it was found that after drug treatment, the main organs of the mice, including the heart, liver, kidney, lung, and spleen, all maintained normal physiological structures (Figure 3B, Figure S4A). Serology assays revealed that aspartate transaminase (AST), alanine transaminase (ALT), alkaline phosphatase (ALP), blood creatinine (CR), and lactate dehydrogenase (LDH) remained within the normal range in all groups (Figure S4B-D). During the first week of drug treatment, there was no significant difference in the levels of BUN among various experimental groups. However, in the second week, the BUN levels in the combination treatment group increased compared with those in the normal saline group. In contrast, the blood urea nitrogen (BUN) levels in the AMNPs treatment group remained within the normal range, which were significantly lower than those in the combination treatment group. This result fully demonstrates that AMNPs have relatively low toxic and side effects (Figure 3C). Moreover, AMNPs showed no significant toxic side effects on the immune system of mice 28 days after administration (Figure S4E). These results confirm that AMNPs have a high level of safety for *in vivo* therapy, and there is no obvious toxicity during the long-term evaluation (within the time range of the graft survival period) of the drug efficacy.

We established a mouse skin transplantation model and evaluated the immunosuppressive effects of the drugs on prolonging the survival period of the grafts. The results showed MMF treatment enhanced the immunotherapeutic effects of ART, prolonging the survival of skin grafts (median survival time [MST] = 20 days). Furthermore, AMNPs treatment achieved better anti-rejection effects than the combination of ART and MMF (median survival time [MST] = 23 days). The immunotherapeutic effect of AMNPs was superior compared to the rapamycin monotherapy (median survival time [MST] = 19 days) reported in our previous evidence (Figure 3D-E) 20. Our treatment strategy also did not show toxicities as evidenced by stable body weight (Figure 3F). Based on the necrotic area score of skin graft rejection 21, AMNPs demonstrated the optimal anti-rejection index, significantly alleviating inflammatory responses such as redness, swelling, and ulceration associated with skin graft rejection (Figure 3G-H).

Haematoxylin & eosin (H&E) staining revealed that the skin grafts of mice administered with saline underwent severe histological rejection. In contrast, the grafts of mice treated with AMNPs exhibited fewer infiltrating inflammatory cells and maintained better vascular integrity. Immunohistochemistry and immunofluorescence staining showed that after treatment with AMNPs, the infiltration of CD4^+^, CD8^+^, and F4/80^+^ cells in the grafts was significantly reduced (Figure 3I, Figure S5A-C). Although there was no significant difference between the AMNPs treatment group and the combination of ART and MMF treatment group on angiogenesis *in vitro* (Figure S6A-B). And treatment with AMNPs significantly enhanced the integrity of both the grafts and blood vessels while reducing the infiltration of inflammatory cells (Figure 3I). This suggests that AMNPs can more effectively inhibit the activation of immune cells, thereby protecting the blood vessels in the allografts from being damaged by immune cells. These results indicate that AMNPs more effectively suppress graft rejection responses, preserving the normal physiological structure of the grafts.

### Immunomodulatory effects of AMNPs on antigen-presenting cells *in vivo*

**Figure 4:**
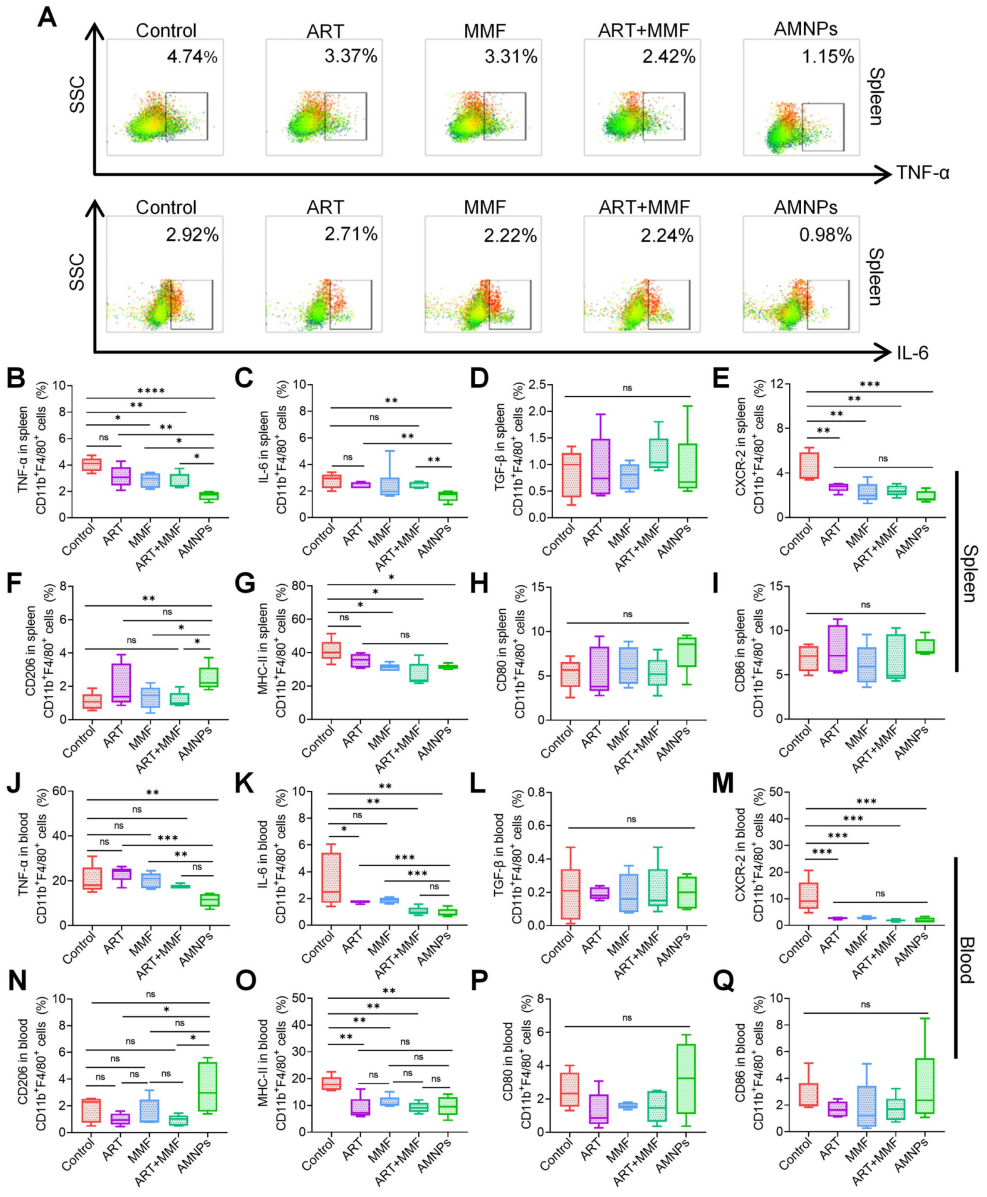
** The effects of ART (17.7 mg/kg), MMF (19.9 mg/kg), the combination of artesunate (17.7 mg/kg) + mycophenolate mofetil (19.9 mg/kg), and AMNPs (ART 17.7 mg/kg; MMF 19.9 mg/kg) on the splenic and peripheral blood antigen-presenting cells in the allogeneic skin transplantation model after 7 - day treatment. (A)** Representative flow cytometry plots showing the expression of TNF-α and IL-6 in splenic CD11b^+^ F4/80^+^ macrophages on POD7. **(B-E)** The TNF-α, IL-6, TGF-β, and CXCR-2 expression in splenic CD11b^+^ F4/80^+^ macrophages (n = 5). **(F-I)** The CD206, MHC-II, CD80, and CD86 expression in splenic CD11b^+^ F4/80^+^ macrophages (n = 5). **(J-M)** The TNF-α, IL-6, TGF-β, CXCR-2 expression in blood CD11b^+^ F4/80^+^ macrophages (n = 5). **(N-Q)** The CD206, MHC-II, CD80, and CD86 expression in blood CD11b^+^ F4/80^+^ macrophages (n = 5). Data are represented as mean ± SD, *p < 0.05, **p < 0.01, ***p < 0.001, ****p < 0.0001, and ns = not significant, p > 0.05.

Macrophages and dendritic cells, as key antigen-presenting cells (APCs), are essential for T cells activation and can orchestrate the immune response against allografts 22. Considering the primary accumulation of AMNPs in splenic macrophages, it is necessary to investigate the immunomodulatory effects of AMNPs on splenic and peripheral blood macrophages. Therefore, a flow cytometry gating strategy diagram is presented (Figure S7A-B). TNF-α, IL-6, and Transforming growth factor-β (TGF-β) are critical cytokines that play critical roles in immune responses and influence macrophage differentiation, thereby regulating the body's inflammatory response to combat infections or transplant rejection 23,24. The combination of ART and MMF led to a notable reduction in the expression of TNF-α in splenic macrophages. Among all treatments, AMNPs exhibited the most potent inhibitory effect on TNF-α production. Although the synergistic inhibitory impact of ART treatment and MMF treatment on IL-6 was not as conspicuous, AMNPs treatment eventually demonstrated a marked inhibitory effect on IL-6 in comparison with the other groups. (Figure 4A-C). In addition, we found that the concentration of IL-6 and TNF-α in the serum of mice decreased after treatment with AMNPs (Figure S7C). However, AMNPs did not significantly inhibit the production of TGF-β in splenic macrophages (Figure 4D). C-X-C motif chemokine receptor type 2 (CXCR-2) is primarily expressed on the surface of macrophages, playing a crucial role in various inflammatory diseases by mediating T cells chemotaxis and activation 25. AMNPs exert a particularly prominent regulatory advantage on CXCR-2 of splenic macrophages (Figure 4E). Additionally, AMNPs demonstrated the strongest inhibitory effect on CXCR-2 expression in BMDMs induced by LPS *in vitro* and effectively reduced the expression of CXCR-2 in the skin graft (Figure S8A-C). AMNPs primarily upregulated the differentiation of M2 macrophages in spleen (Figure 4F). In all groups, there was no significant impact on the expression of CD80 and CD86 in splenic macrophages. However, the expression of MHC-II in splenic macrophages was significantly decreased in the AMNPs treatment group (Figure 4G-I).

Furthermore, we analyzed the effect of AMNPs on macrophages in the circulating blood and observed similar effects. AMNPs significantly reduced the expression of IL-6 and TNF-α in peripheral blood macrophages (Figure 4J-K). There was no significant impact on the expression of TGF-β in blood macrophages. However, CXCR-2 expression in blood macrophages consistently declined in each treatment (Figure 4L-M). Interestingly, following AMNPs treatment, macrophages in the bloodstream were significantly induced to differentiate into the M2 phenotype (Figure 4N). Moreover, after AMNPs treatment, the expression of MHC-II in macrophages was significantly reduced, but there was no significant effect on the expression of CD80 and CD86 on blood macrophages (Figure 4O-Q). AMNPs also had a certain effect on dendritic cells. Our results showed that AMNPs treatment only reduced CD86 expression in peripheral blood dendritic cells, while having no effect on the infiltration of macrophages and dendritic cells in the spleens or peripheral blood (Figure S9A-D). These results indicate that AMNPs primarily reduce the expression of TNF-α and IL-6 in macrophages and promote the differentiation of M2 phenotype macrophages, thereby exerting their immunosuppressive effects *in vivo*.

### Adoptive transfer of macrophages extends graft survival

**Figure 5:**
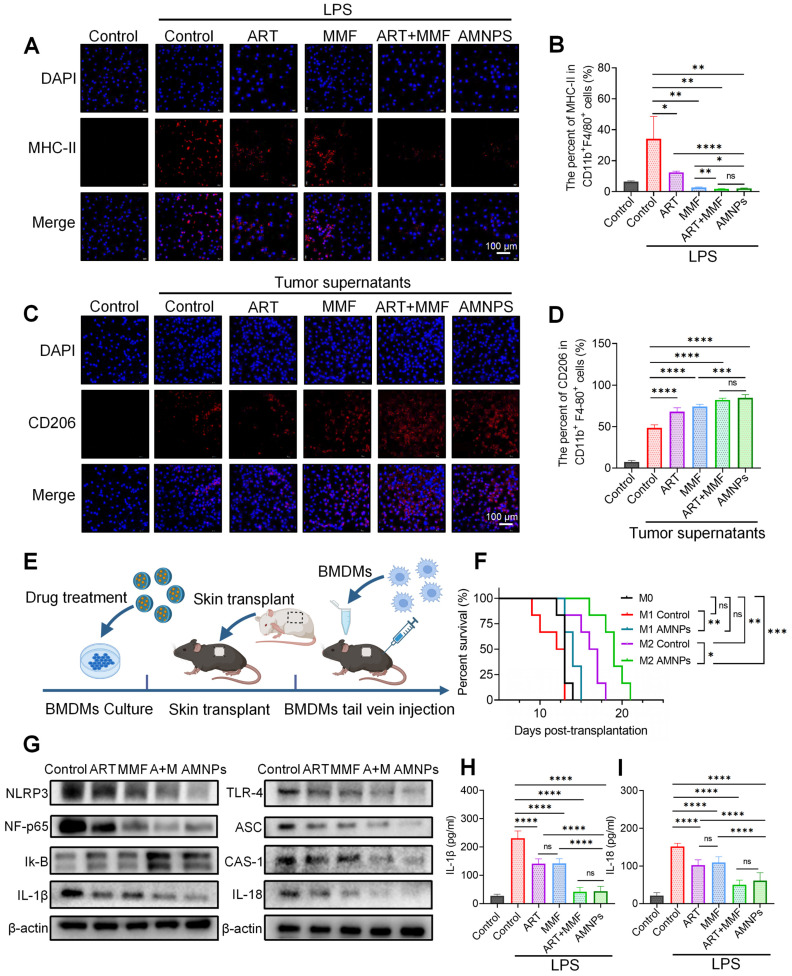
** The effects of ART (10 μM), MMF (10 μM), the combination of ART + MMF (each at 10 μM), and AMNPs (10 μM) on BMDMs differentiation *in vitro* and the macrophage adoptive transfer experiment. (A)** The validation of MHC-II expression in BMDMs by immunofluorescence analysis. Scale bar: 100 μm (n = 3). **(B)** The MHC-II expression in BMDMs *in vitro* by flow cytometry analysis (n = 4). **(C)** The validation of CD206 expression in BMDMs by immunofluorescence analysis. Scale bar: 100 μm (n = 3). **(D)** The CD206 expression in BMDMs *in vitro* by flow cytometry analysis (n = 4). **(E)** Schematic diagram of BMDM adoptive transfer into C57BL/6 mice. **(F)** Skin graft survival analysis in C57BL/6 mice following adoptive transfer of M0, M1, or M2 type macrophage (n = 6). **(G)** The representative western blots analysis in the TLR-4/NF-κB/NLRP3 pathway of BMDM (n = 3). **(H, I)** The protein concentrations of IL-1β and IL-18 in the supernatant of BMDMs determined by ELISA assay (n = 6). Data are represented as mean ± SD, *p < 0.05, **p < 0.01, ***p < 0.001, ****p < 0.0001, and ns = not significant, p > 0.05.

Building on the *in vivo* effects of AMNPs on macrophage polarization, we performed *in vitro* experiments to confirm its impact on the polarization of BMDMs. Immunofluorescence analysis demonstrated that LPS stimulation significantly increased the expression of MHC-II in BMDMs, indicating differentiation of macrophages toward the M1 phenotype. ART exhibited a more potent inhibition of M1 macrophage differentiation compared to MMF, and the combination of MMF and ART treatment further enhanced its immunosuppressive effects. The combination treatment and AMNPs treatment effectively suppressed M1 macrophage differentiation *in vitro* (Figure 5A). We further verified the results of immunofluorescence through flow cytometry and obtained consistent results (Figure 5B). These results further revealed the immunomodulatory effect of AMNPs on macrophages, and verified the experimental results of AMNPs' impact on macrophage differentiation by targeting the spleen.

Next, we used tumor supernatant from Hepa1-6 cells to induce the differentiation of macrophages toward the M2 phenotype, and the expression of CD206 in BMDMs was significantly upregulated compared to the untreated group. Similarly, Immunofluorescence and flow cytometry analyses indicated that AMNPs treatment effectively promoted the differentiation of macrophages toward the M2 phenotype (Figure 5C-D). These results indicate that AMNPs could effectively shift pro-inflammatory M1 macrophages toward the differentiation of anti-inflammatory M2 macrophages *in vitro*.

We further inferred that the adoptive transfer of M1 and M2 macrophages in a mouse skin transplantation model may affect the transplantation immune environment and influence the survival period of the grafts. We intravenously transferred the AMNPs-induced M1 and M2 macrophages into C57BL/6 mice that were receiving BALB/c skin grafts, using naïve M0 macrophages, which had not been pre-treated with LPS or tumor supernatants, as controls (Figure 5E). The survival period of mice receiving adoptively transferred AMNPs pretreated M1 macrophages (median survival time [MST] = 14 days) was significantly prolonged compared to the group that received untreated M1 macrophages (median survival time [MST] = 12.5 days). However, there was no significant difference compared to the M0 macrophage transfer group (median survival time [MST] = 13 days). This may be due to the activation of M0 macrophages by the immune environment of the skin graft, leading to their differentiation into M1 macrophages, which reduced the difference in the amount of effective M1 macrophages between the AMNP-treated M1 macrophage group and the M0 macrophage transfer group *in vivo*. Interestingly, the survival of mice in the group receiving AMNP-treated M2 macrophage (median survival time [MST] = 19 days) adoptive transfer was significantly extended compared to the group with untreated M2 macrophages (median survival time [MST] = 16.7 days), with both groups showing a notably longer survival than the M0 macrophage transfer group (Figure 5F). This study shows that treatment with AMNPs can induce macrophages to polarize towards the M2 phenotype, exerting an immunosuppressive effect and alleviating transplant rejection.

Macrophages play an important role in the immune environment. We investigated whether AMNPs have an impact on the gene transcription level of macrophages and conducted transcriptome sequencing of BMDMs. As a result, Kyoto Encyclopedia of Genes and Genomes (KEGG) analysis results show that after treatment with AMNPs, the differentially expressed genes related to pathways such as Toll-like receptors, nuclear factor kappa B (NF-κB), TNF, and those related to interaction with T cells in macrophages show a significant downward trend. Gene Set Enrichment Analysis (GSEA) revealed a significant downregulation of the mRNA expression of inflammatory pathways and Toll-like receptors in macrophages (Figure S10A-C). Toll-like receptor-4 (TLR-4) activates the nuclear factor kappa B (NF-κB)/NOD-like receptor family pyrin domain containing 3 (NLRP3) pathways, which promotes the maturation and release of interleukin-1β (IL-1β) and interleukin-18 (IL-18), subsequently enhancing the polarization of M1 macrophages 26,27. The effect of AMNPs on TLR-4/NF-κB/NLRP3 signalling was furthermore determined via Western blot analysis. The results show that MMF enhances the inhibitory effect of ART on the TLR-4/NF-κB/NLRP3 pathway. Furthermore, the Western blot results indicate a downregulation of TLR-4, NF-κB, NLRP3, Caspase-1 (CAS-1), apoptosis-associated speck-like protein (ASC), IL-1β, and IL-18 protein expression, while Inhibitor of Nuclear Factor-κB (Iκ-B) protein expression is upregulated in BMDMs after AMNPs treatment (Figure 5G). We investigated whether AMNPs could affect the secretion of pro-inflammatory cytokines in BMDMs and found that the levels of IL-1β and IL-18 in the supernatants of BMDMs treated with AMNPs were also significantly reduced (Figure 5H-I). These findings indicate that AMNPs can modulate the TLR-4/NF-κB/NLRP3 pathway in BMDMs, thereby regulating the pro-inflammatory phenotype and differentiation of BMDMs, and enhancing the survival of grafts after BMDMs reinfusion.

### Regulation of T cells differentiation following the AMNPs administration

**Figure 6:**
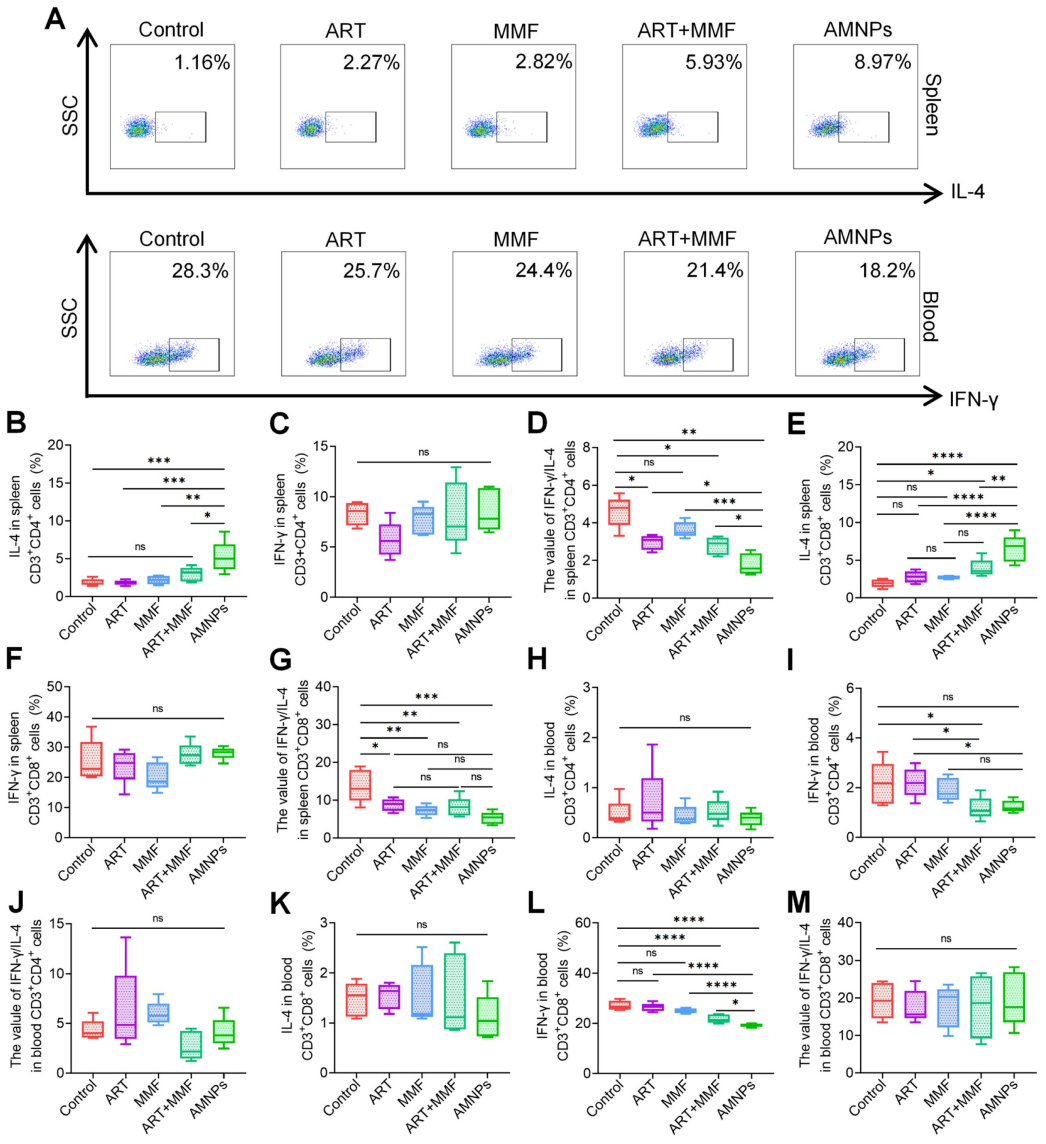
** The effects of ART (17.7 mg/kg), MMF (19.9 mg/kg), the combination of artesunate (17.7 mg/kg) and mycophenolate mofetil (19.9 mg/kg), and AMNPs (ART: 17.7 mg/kg; MMF:19.9 mg/kg) on the splenic and peripheral blood T cells in the allogeneic skin transplantation model after 7 days treatment. (A)** Representative flow cytometry plots showing the expression of IL-4 in splenic CD3^+^ CD8^+^ T cells and IFN-γ in blood CD3^+^ CD8^+^ T cells on POD7. **(B-D)** The expression of IL-4, IFN-γ, and IFN-γ/IL-4 ratio in splenic CD3^+^ CD4^+^ T cells (n = 5). **(E-G)** The expression of IL-4, IFN-γ, and IFN-γ/IL-4 ratio in splenic CD3^+^ CD8^+^ T cells (n = 5). **(H-J)** The expression of IL-4, IFN-γ, and IFN-γ/IL-4 ratio in blood CD3^+^ CD4^+^ T cells (n = 5). **(K-M)** The expression of IL-4, IFN-γ, and IFN-γ/IL-4 ratio in blood CD3^+^ CD8^+^ T cells (n = 5). Data are represented as mean ± SD, *p < 0.05, **p < 0.01, ***p < 0.001, ****p < 0.0001, and ns = not significant, p > 0.05.

*In vitro*, we observed that AMNPs significantly reduced the interferon-gamma (IFN-γ)/(interleukin 4) IL-4 values in CD3^+^ CD4^+^ T cells, thereby modulating the Th1/Th2 balance (Figure S11A-B). Since AMNPs predominantly accumulate in macrophages, with only a small fraction taken up by T cells, we further isolated mononuclear cells from the splenic and peripheral blood to investigate the immunomodulatory effects of AMNPs on T cells *in vivo*, and constructed flow cytometry gating strategy diagrams to illustrate the process (Figure S12A). We found that AMNPs indeed reduced IFN-γ levels and increased IL-4 levels in T cells *in vivo* as shown in the representative flow cytometry analysis diagram (Figure 6A). AMNPs primarily enhanced the expression of IL-4 in splenic CD3^+^ CD4^+^ T cells, effectively shifting the balance between Th1 and Th2 cells (Figure 6B-D). This modulation influences the immune response related to transplant rejection. AMNPs promote a Th2-dominant environment, mitigate the inflammatory responses typically associated with transplant rejection, and improve graft acceptance. MMF treatment enhanced the upregulation of IL-4 expression in splenic CD3^+^ CD8^+^ T cells induced by ART treatment, while AMNPs exhibited a superior regulatory effect in this regard. And both MMF and AMNPs treatment groups effectively downregulated the splenic Th1/Th2 balance compared to the control group (Figure 6E-G).

CD3^+^ CD8^+^ T cells can recognize foreign antigens present in the graft, which can trigger a rejection response. In contrast, reduced CD8^+^ T cells activity may contribute to enhanced graft tolerance 28. In peripheral blood, AMNPs primarily downregulated the IFN-γ levels in both CD3^+^CD4^+^ T cells and CD3^+^CD8^+^T cells, and AMNPs treatment group demonstrated a more pronounced effect on regulating IFN-γ levels in CD3^+^ CD8^+^ T cells compared to the combination of ART and MMF treatment group (Figure 6H-M). Interestingly, the percent of CD4^+^ Tregs were increased and the proportion of effector CD3^+^ CD4^+^T cells in peripheral blood were significantly decreased following AMNPs treatment compared to other treatment group (Figure S12B-D). Tregs inhibit the activation and proliferation of CD3^+^ CD8^+^ T cells, as well as the production of IFN-γ derived from them. This suppression reduces excessive immune responses and helps prevent damage to self-tissues 29. These elucidate why IFN-γ levels in CD3^+^ CD8^+^ T cells were effectively reduced in peripheral blood rather than in the spleens, thereby offering protection against grafts damage. AMNPs significantly inhibited the proliferation of CD4^+^ and CD8^+^ T cells in the CFSE proliferation assays (Figure S13A-B). These findings indicate that AMNPs possess the capacity to effectively regulate the proliferation and differentiation of T cells, consequently exerting an impact on the immune environment.

### AMNPs target macrophages and indirectly affect T cells proliferation and activation

**Figure 7:**
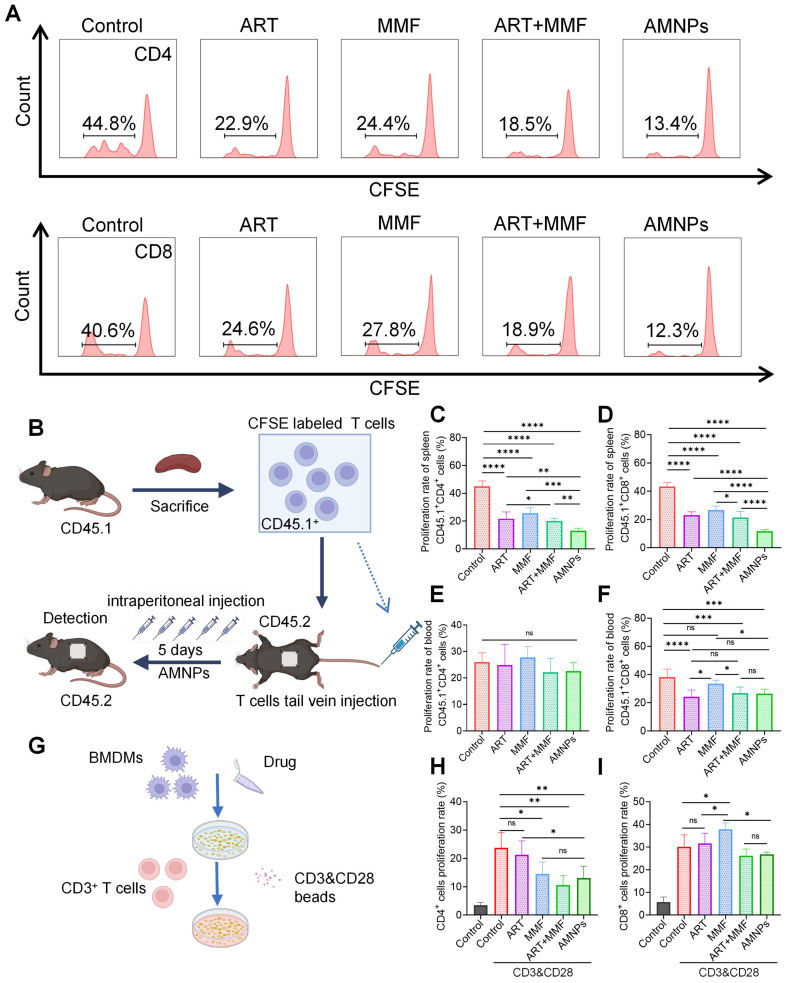
In allogeneic skin transplantation in CD45.2 C57BL/6 mice, followed by adoptive transfer of CD45.1^+^ T cells and treated with ART (17.7 mg/kg), MMF (19.9 mg/kg), the combination of artesunate (17.7 mg/kg) and mycophenolate mofetil (19.9 mg/kg), or AMNPs (ART, 17.7 mg/kg; MMF, 19.9 mg/kg) for 5 days. **(A)** Representative flow cytometry histogram showing the proliferation rate of splenic CD45.1^+^ CD3^+^ T cells and CD45.1^+^ CD8^+^ T cells. **(B)** Schematic diagram of the T cells adoptive transfer proliferation experiment and drug administration *in vivo*. **(C, D)** Proliferation rate of CD45.1^+^ CD3^+^ CD4^+^ T cells and CD45.1^+^ CD3^+^ CD8^+^ T cells in the splenic of C57BL/6 CD45.2 mice (n = 6). **(E, F)** Proliferation rate of CD45.1^+^ CD3^+^ T cells and CD45.1^+^ CD8^+^ T cells in the blood (n = 6). **(G)** The co-culture sketch map of BMDMs and CD3^+^ T cells treated with ART, MMF, the combination of ART and MMF, or AMNPs at 10 μM. **(H-J)** Proliferation rate of CD3^+^ CD4^+^T cells and CD3^+^ CD8^+^ T cells in the co-culture system (n = 5). Data are represented as mean ± SD, *p < 0.05, **p < 0.01, ***p < 0.001, ****p < 0.0001, and ns = not significant, p > 0.05.

To preferably evaluate the inhibitory effects of AMNPs on T cells proliferation *in vivo*, we established a model where C57BL/6 CD45.2 mice received skin grafts from BALB/c mice. The mononuclear cells from the spleens of C57BL/6 CD45.1 mice were isolated, labeled with CFSE, and then adoptively transferred into C57BL/6 CD45.2 mice. Subsequently, drug treatment was administered to the recipient mice for 5 days. On Postoperative day 5 (POD5), we harvested the grafts and, through immunofluorescence experiments, found that the infiltration of CFSE-labeled mononuclear cells in the grafts from the AMNPs treatment group was significantly reduced compared to the ART and MMF treatment groups (Figure S14A). This suggests that AMNPs regulate the immune response, limiting the proliferation of immune cells within the host, which in turn reduces the number of these cells migrating to the graft. This effect contributes to improving the immune environment of the skin graft and enhancing graft survival. We further analyzed the proliferation of adoptively transferred T cells in the spleens and peripheral blood using flow cytometry, presenting the flow cytometry gating strategy and representative peak plots (Figure 7A-B, Figure S14B). Although AMNPs primarily accumulated in splenic macrophages with less accumulation in T cells, AMNPs treatment group still significantly reduced the proliferation rates of CD45.1^+^CD3^+^CD4^+^ and CD45.1^+^CD3^+^CD8^+^ T cells in the spleens compared to the combination of ART and MMF treatment group (Figure 7C-D). However, despite the fact that treatment with AMNPs led to a reduction in the proliferation rate of CD45.1^+^CD8^+^ T cells in peripheral blood, no significant difference was observed when compared to the combination of ART and MMF treatment group (Figure 7E-F). M1 macrophages promote T cells proliferation and induce Th1 differentiation via producing various pro-inflammatory cytokines, such as IL-6 and TNF-α 30. In contrast, M2 macrophages excessively secrete immunosuppressive cytokines such as interleukin 10 (IL-10) and TGF-β, which help induce the differentiation of CD3^+^ CD4^+^ T cells into Tregs and suppress the activation of Th1 and Th17 cells 31. Therefore, we speculate that AMNPs are primarily phagocytosed by splenic macrophages, and by regulating the immune phenotype of these macrophages, they indirectly affect the proliferation and activation of T cells. This results in a significant impact on the proliferation of splenic T cells, while showing a relatively lesser effect on T cells proliferation in peripheral blood. Next, we cultured M1 type BMDMs and treated them with AMNPs, followed by co-culturing with splenic CD3^+^ T cells to investigate the impact of macrophages on T cells proliferation (Figure 7G). The results indicated that co-culturing M1 macrophages with T cells significantly promoted T cells proliferation and activation. Through the co-culture experiment of macrophages and T cells, we found that macrophages treated with AMNPs significantly reduced the immunoactivating effects of CD3^+^ CD4^+^ and CD3^+^ CD8^+^ T cells (Figure 7H-I). Therefore, the treatment regimen of AMNPs in the transplantation model may target macrophages and indirectly affect the proliferation and activation of T cells.

### Macrophage clearance experiment verifies the regulatory effect of macrophages on T cells

**Figure 8:**
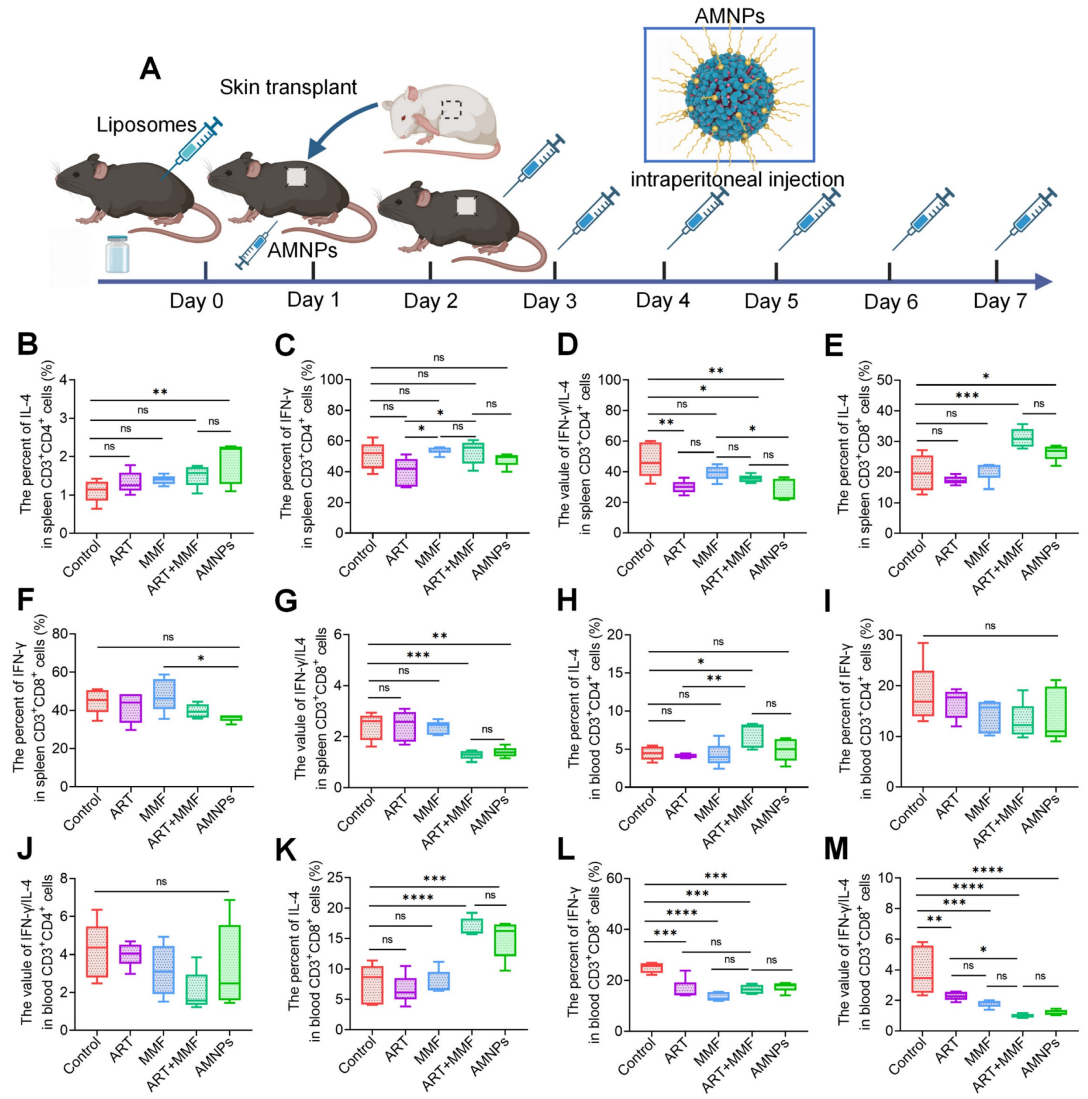
Clodronate liposomes were used to deplete the macrophages of the allogeneic skin transplantation C57BL/6 mice, and peripheral blood and spleens were collected following 7 days of ART (17.7 mg/kg), MMF (19.9 mg/kg), the combination of artesunate (17.7 mg/kg) and mycophenolate mofetil (19.9 mg/kg), and AMNPs (ART: 17.7 mg/kg; MMF: 19.9 mg/kg) treatment to analysis T cells differentiation via flow cytometry. **(A)** Schematic diagram of the Macrophage clodronate liposomes depletion assay and drug administration *in vivo*. **(B-D)** The IL-4, IFN-γ, and IFN-γ/IL-4 expression of splenic CD3^+^ CD4^+^ T cells (n = 5). **(E-G)** The IL-4, IFN-γ, and IFN-γ/IL-4 expression of splenic CD3^+^ CD8^+^ T cells (n = 5). **(H-J)** The IL-4, IFN-γ, and IFN-γ/IL-4 expression of blood CD3^+^ CD4^+^ T cells (n = 5). **(K-M)** The IL-4, IFN-γ, and IFN-γ/IL-4 expression of blood CD3^+^ CD8^+^ T cells (n = 5). Data are represented as mean ± SD, *p < 0.05, **p < 0.01, ***p < 0.001, ****p < 0.0001, and ns = not significant, p > 0.05.

To further determine the crucial role of macrophages that phagocytosed AMNPs in regulating T cells, we used clodronate liposomes to deplete macrophages in C57BL/6 mice before they received BALB/c skin grafts. C57BL/6 mice were treated with ART, MMF, the combination of ART and MMF, or AMNPs. To evaluate the impact of drug treatment on T-cell differentiation, mononuclear cells were isolated from splenic and peripheral blood samples (Figure 8A). Following AMNPs treatment, the IL-4 levels in splenic CD3^+^ CD4^+^ T cells showed a slight decrease, while there was no significant effect on IFN-γ levels (Figure 8B-C). Similarly, AMNPs treatment decreased the ratio of IFN-γ/IL-4 in splenic CD3^+^ CD4^+^ T cells significantly compared to the control group; Previously observed significant differences compared to the combination of ART and MMF treatment group vanished (Figure 8D). This indicates that the accumulation of AMNPs in macrophages plays a crucial role in regulating T cells activity. The IL-4 levels in splenic CD3^+^ CD8^+^ T cells also exhibited a similar trend to that observed in CD3^+^ CD8^+^ T cells following AMNPs treatment (Figure 8E-F). Furthermore, there was no significant difference in the ratio of IFN-γ/IL-4 between the AMNPs treatment group and the combination of ART and MMF treatment group (Figure 8G). Following the depletion of macrophages, the targeting and accumulation advantage of AMNPs on macrophages vanished, thereby weakening their regulatory effect on T cells, indicating that macrophages play an important role in T cell differentiation.

There were no significant differences in the levels of IFN-γ and IL-4 as well as the ratio of IFN-γ /IL-4 in blood CD3^+^ CD4^+^ T cells between the AMNPs treatment group and the combination of ART and MMF treatment group. However, the expression of IL-4 in blood CD3^+^ CD4^+^ cells in the combination of ART and MMF treatment group was elevated compared to the control group (Figure 8H-J). The combination of ART and MMF treatment group and AMNPs treatment group showed no significant difference in regulating the Tc1/Tc17 balance of blood CD3^+^ CD8^+^ T cells. However, MMF treatment enhanced the anti-inflammatory differentiation effect of ART treatment in blood CD3^+^ CD8^+^ T cells. Compared with monotherapy, the combination of ART and MMF treatment demonstrated a more pronounced downward trend the ratio of IFN-γ/IL-4 in blood CD3^+^ CD8^+^ T cells, indicating a enhanced anti-inflammatory effect (Figure 8K-M). This observation might be ascribed to the fact that, in the context of acute rejection, blood CD3^+^ CD8^+^ T cells exhibit a greater propensity for undergoing substantial alterations compared with CD3^+^ CD4^+^ T cells. Given that CD3^+^ CD8^+^ T cells directly participate in cytotoxic attacks against the graft, they contribute significantly to tissue damage. The increased activity and proliferation of blood CD3^+^ CD8^+^ T cells in this context makes them more susceptible to regulation via CD4^+^ Tregs 32. Overall, these data indicate that AMNPs direct macrophage differentiation and pro-inflammatory phenotypes, further attenuating T cell-mediated rejection of the graft and reducing graft damage. These findings reveal the potential role of AMNPs in the regulation of transplant immunity.

## Discussion

Despite considerable advancements in perioperative management over the past few decades, allograft rejection remains one of the most prevalent and challenging complications following organ transplantation. This immune response can progressively deteriorate graft function, and in serious cases, result in total graft failure, significantly compromising the recipient's survival and quality of life 33. Conventional immunosuppressive agents commonly employed in clinical practice frequently encounter challenges such as poor solubility and difficulties in effectively targeting immune organs. Although high doses of immunosuppressants are frequently administered orally following organ transplantation to ensure adequate immunosuppressive efficacy, the risk of drug-related adverse reactions among recipients significantly rises 34,35. Calcineurin inhibitors, acting as the cornerstone of immunosuppressive regimens after organ transplantation, are recognized as significant factors contributing to renal dysfunction. They may also increase the risk of Hepatitis C Virus (HCV) reinfection and tumor recurrence 36. In clinical practice, MMF is commonly used in conjunction with other immunosuppressive agents, such as cyclosporine and prednisone, to enhance immunosuppressive efficacy and mitigate the risk of rejection. However, the long-term use of MMF can result in various side effects, including gastrointestinal discomfort and leukopenia 37.

ART stands as the drug of choice for the clinical management of severe malaria cases. In contrast to traditional immunosuppressive agents, ART is recognized for its excellent human tolerance and minimal side effects 38. Recent studies have explored the potential of ART in treating autoimmune diseases by modulating immune responses and reducing immune rejection reactions in mouse heart transplantation model 39,40. Therefore, we investigated whether MMF could enhance the immunosuppressive efficacy of artesunate, reduce drug toxicity, and improve the recipient's tolerance to the treatment. We modified ART and MMF through an esterification reaction, enabling this prodrug to self-assemble into AMNPs in aqueous solution. Surprisingly, systemically administered AMNPs were capable of accumulating in macrophages within the spleens. Further research indicated that AMNPs could suppress T cell proliferation and pro-inflammatory differentiation by modulating key macrophage cytokines, namely TNF-α and IL-6. The anti-rejection effects of AMNPs were further validated in an allogeneic mouse skin transplantation model. In comparison with rapamycin, a widely used immunosuppressant in clinical organ transplantation, AMNPs more effectively inhibited the immune rejection reaction and prolonged the survival period of the grafts (23 days vs. 19 days).

Artesunate, a semi-synthetic derivative of artemisinin extracted from the traditional Chinese herb Artemisia annua (sweet wormwood) and then further modified, is widely employed for the treatment of malaria 41. Recently, ART has exhibited powerful antimalarial activity and has become the preferred treatment option for severe malaria, especially cerebral malaria due to its rapid onset and low toxicity 42. In addition to its antimalarial properties, artesunate has shown potential in treating autoimmune disorders and alleviating chronic immune rejection after transplantation through modulating immune responses 43,44. Therefore, MMF may synergistically inhibit the immune rejection of grafts with artesunate and reduce the drug's toxic and side effects. And we chemically modified ART with MMF to develop AMNPs, and validated the immunosuppressive efficacy of AMNPs via the acute rejection mouse skin transplantation model. CD8^+^ T cells can directly recognize and kill graft cells by identifying the antigens presented on the surface of the graft. In addition, CD8^+^ T cells secrete various cytokines (TNF-α and IFN-γ) to enhance the cytotoxicity of CD8^+^ T cells and exacerbate the rejection response 45. Moreover, the inflammatory response triggered by CD8^+^ T cells may lead to vascular damage, consequently reducing graft survival rates 46. Our results demonstrated that AMNPs effectively increase blood drug concentration through their sustained-release mechanism, enabling continuous drug release in the body and maintaining stable therapeutic levels. The administration of AMNPs further modulates immune cells in the bloodstream, particularly reducing the function of CD8^+^ T cells. Skin transplantation, a model of acute immune rejection, is characterized by local redness and swelling within a few days after transplantation, tissue necrosis, proliferation of CD8^+^ T cells, as well as vasculitis or damage to endothelial cells 47. The results indicate that AMNPs treatment remarkably reduces the infiltration of F4/80^+^ and CD8^+^ T cells in the grafts, while maintaining the grafts' optimal physiological structure and vascular integrity. Our results show that AMNPs regulate the function of macrophages and inhibit the pro-inflammatory activation of macrophages by regulating the secretion of various inflammation-related cytokines. AMNPs effectively reduce the secretion of pro-inflammatory cytokines like TNF-α and IL-6, thus alleviating inflammatory responses. In general, AMNPs regulate macrophage function, reduces tissue damage, and assists in maintaining the vascular and tissue integrity of the graft, thus supporting tissue repair and functional maintenance after transplantation 48.

We determined the safe dosage of the drug in accordance with relevant studies 49-51. In the model of allogeneic heart transplantation in rats, it has been reported that the dosage of MMF used was 20 mg/kg 52. In AMNPs, ART and MMF are linked via esterification at a 1:1 ratio. Given the 1:1 molar ratio of MMF to ART in the AMNPs prodrugs, we determined the dosage of AMNPs, and the corresponding dosages of ART and MMF are 17.7 mg/kg and approximately 20 mg/kg respectively. After skin transplantation, T cells and other immune cells were activated and differentiated on POD7 53. At this time point, the immunomodulatory effect of AMNPs on immune cells can be observed. However, the rejection phenotype of T cells in skin allografts takes about 9 days to show more obvious changes. Therefore, changes in immune cells generally occur earlier than when the differences in the transplantation rejection phenotype can be observed. Studies on heart transplantation models have demonstrated that obvious activation of immune cells in the graft can be observed on POD7 54. This shows that immune cells can indeed activate the immune response seven days after being stimulated by allogeneic graft antigens. Hence, we chosen to conduct the detection on POD7. The administration strategy involves intraperitoneal injection of ART, MMF, the combination of ART and MMF, and AMNPs for the treatment of mouse allogeneic skin transplantation. Macrophages are generally regarded as scavengers *in vivo*. In the context of cancer treatment involving nanodrugs, they are sometimes considered a drawback. However, this characteristic may bring benefits in the field of organ transplantation therapy 55. In organ transplantation, monocytes and macrophages originating from peripheral blood and pre-existing organ tissues are the primary cells that infiltrate the implanted allografts 56. Additionally, it is widely held that macrophages, rather than T cells, are more likely to capture drug-loaded particles. Therefore, we hypothesized that therapeutic nanoparticles effectively taken up by macrophages might help alleviate allogeneic transplant rejection 57. Indeed, we found that macrophages successfully captured AMNPs *in vitro*, and this capture process facilitated their polarization into M2 macrophages, resulting in reduced expression of TNF-α and IL-6. M2 macrophages are typically considered anti-inflammatory and immunosuppressive effector cells that influence T cell proliferation and differentiation by secreting specific cytokines 58, thereby mitigating excessive immune responses and promoting tissue repair and regeneration. Interestingly, our study revealed that after macrophage depletion, the regulatory effect of AMNPs on the Th1/Th17 balance in T cells was diminished. This indicates that macrophage capture of AMNPs alters the pro-inflammatory phenotype and further inhibits the pro-inflammatory differentiation of T cells. Our study demonstrated the immunoregulatory role of macrophages on T cells through *in vitro* co-culture experiments. Macrophages treated with AMNPs exhibited reduced T cells proliferation capacity *in vitro* compared to the control group. We performed adoptive transfer of CFSE labeled T cells into C57BL/6 mice. Subsequently, we observed a more significant inhibition of T cells proliferation in the spleens as compared to that in the blood. This finding indicates that M2 macrophages guided by AMNPs exerted a further inhibitory effect on T cells proliferation. These results evidence the critical role of macrophages that have phagocytosed AMNPs in regulating the immune environment dominated by CD8^+^ T cells during immune rejection.

Following transplantation, immune cells in the peripheral blood (such as T cells, B cells, and macrophages) rapidly migrate to the transplant site through the bloodstream, influenced by chemotactic factors and regulated by immune signals 59. Macrophages express CXCR-2, which facilitates the release of chemokines such as CXCL-1 and CXCL-2 to recruit T cells to sites of inflammation or infection 60. After treatment with AMNPs, we observed a significant reduction in both the mRNA levels of CXCR-2 in the grafts and the expression of CXCR-2 in macrophages, leading to a decrease in the number of T cells recruited to the skin graft. Tregs in the immune system secrete inhibitory cytokines that reduce the expression of IFN-γ in CD8^+^ T cells, thereby preventing the overactivation of CD8^+^ T cells and minimizing potential tissue damage and autoimmune responses 61. And AMNPs treatment promoted the differentiation of M2 macrophages in peripheral blood and increased the ratio of CD4^+^ Tregs to effector CD4^+^T cells, thereby further reducing the level of IFN-γ in CD8^+^ T cells. Previous studies have reported that reinfusing macrophages can regulate the body's immune microenvironment, thus achieving a certain immunotherapeutic effect 62. We further investigated whether the adoptive transfer of M1 and M2 macrophages into skin-transplant mice would affect the progression of graft immune rejection. After treatment with AMNPs, macrophages are able to shift towards the M2 phenotype, and when reintroduced into mice, they significantly prolong graft survival. We further investigated the molecular pathway of AMNPs in macrophage immunoregulation and performed transcriptome sequencing on AMNP-treated macrophages. GSEA showed a significant downregulation of the mRNA expression of inflammatory pathways and Toll-like receptors. Combination therapy with MMF and tacrolimus can significantly inhibit the TLR-4/NF-κB/NLRP3 pathway 63,64. There have been relevant studies that have proven the research on the effects of Artesunate and MMF on the TLR-4 pathway 65,66. Therefore, we hypothesize that AMNPs exert their immunosuppressive functions through the TLR-4/NF-κB/NLRP3 signaling pathway. The results showed that the combined use of ART and MMF more significantly reduced the activation of the TLR-4/NF-κB/NLRP3 signaling pathway in BMDMs. Furthermore, the ability of macrophages to secrete IL-1β and IL-18 was also effectively reduced. The activation of the NLRP3 pathway promotes the secretion of IL-1β and IL-18, thereby facilitating the polarization of macrophages toward the M1 type. Excessive activation of the NLRP3 pathway can inhibit the polarization of M2 macrophages 67. Therefore, AMNPs modulate T cell proliferation and pro-inflammatory differentiation after the adoptive transfer of M2 macrophages by promoting M2 macrophage polarization and reducing the secretion of pro-inflammatory factors involved in the NLRP3 pathway. This extends grafts survival and preserves the functional status of the grafts.

## Conclusions

**Figure 9:**
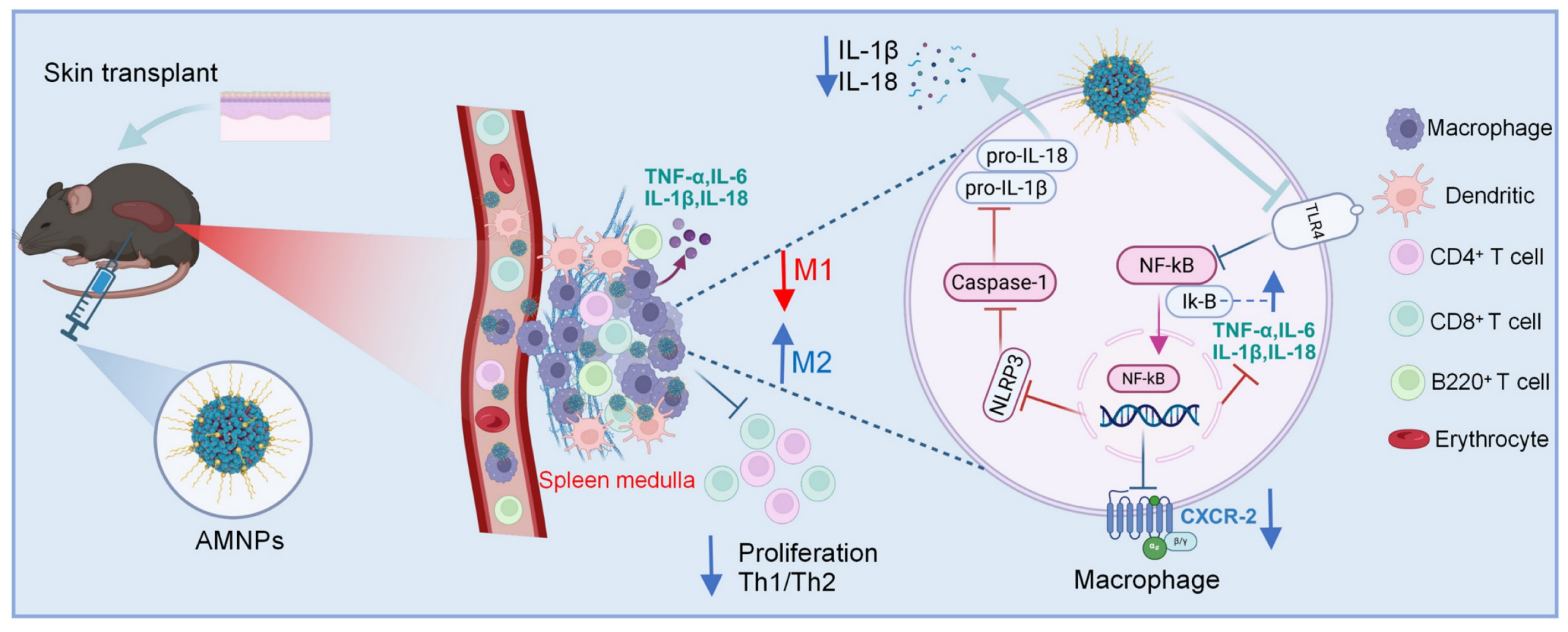
** (Graphical Abstract) The schematic illustration of the nanotherapeutic strategy shows the chemical modification of ART with MMF, resulting in the self-assembly of nanoparticles.** AMNPs exhibit targeting capabilities for immune organs, enhancing drug delivery efficiency and enabling specific immunotherapy for immune cell subpopulations. The therapeutic strategy of AMNPs achieves long-term grafts survival by immunoregulating both macrophages and T cells.

Overall, we carried out molecular editing of artesunate and mycophenolate mofetil and developed AMNPs through nano-assembly. These nanoparticles mainly accumulate in the spleens, significantly enhancing bioavailability. Compared with the combined treatment of ART and MMF, AMNPs exhibit powerful immunosuppressive effects and enhanced biocompatibility. In mouse skin transplantation models, the administration of AMNPs significantly mitigated the risk of rejection and prolonged the survival time of the graft. Mechanistically, AMNPs primarily target macrophages, inducing their differentiation into anti-inflammatory M2 macrophages. This, in turn, enhances the T cell mediated Type 2 immune response, suppresses Type 1 immune responses, and increases the proportion of CD4^+^ Tregs in peripheral blood, effectively inhibiting the cytotoxic function of CD8^+^ T cells. In summary, we have developed a novel molecular inhibitor and its clinically relevant formulation, which could serve as a new immunosuppressive agent for preventing acute rejection (Figure 9). Given the urgent clinical need for novel immunosuppressive therapies, our strategy may offer a promising new therapeutic option.

## Materials and Methods

### Synthesis and characterization of AMNPs prodrugs

AMNPs prodrugs were prepared by modifying artesunate and mycophenolate mofetil through an esterification reaction and were characterized using NMR and MS. The synthetic protocols are provided in the Supplementary Information.

### Preparation of self-assembled ART-MMF nanoparticles

The AMNPs were prepared using a solvent evaporation followed by ultrasonication technique. AMNPs prodrug and DSPE-PEG_2k_ were dissolved in 20% acetone, and under ultrasonication, they self-assembled into nanoparticles in deionized water. Subsequently, acetone was slowly removed by rotary evaporation. Then, dynamic light scattering (DLS, Malvern Nano-ZS90, Malvern, UK) was used to analyze the D_H_, PDI, and zeta potential of the AMNPs. The morphology of the AMNPs was revealed by transmission electron microscopy (TEM, Tecnai G2, Philips, Netherlands).

### Cell culture

Splenic mononuclear cells from the spleens of C57BL/6 mice (male, 6 weeks old, weighing 20-25 g) were cultured in RPMI-1640 (Cat# 11875085, Gibco, USA) supplemented with 10% FBS (Cat# 10091148, Gibco, USA), and interleukin 2 (IL-2) (30 IU/mL). The bone marrow cavity of the hind leg of C57BL/6 mice was flushed with serum-free 1640 medium. The collected bone marrow cells were cultured in 12-well plates with RPMI-1640 medium containing 10% FBS and 40 ng/mL M-CSF. The human umbilical vein endothelial cell (HUVEC) was purchased from the American Type Culture Collection and maintained in Dulbecco's Modified Eagle Medium (Cat# 11875085, Gibco, USA) containing 10% FBS (Manassas, VA, USA). Murine liver cancer cell line Hepa1-6 (RRID: CVCL_0327) was obtained from China Infrastructure of Cell Line Resources (Beijing, China) maintained in RPMI-1640 containing 10% FBS. The cell lines were authenticated by Short Tandem Repeat (STR) tests. All cells grew in media with 100 μg/mL penicillin and 100 μg/mL streptomycin (Cat# 15070063, Gibco, USA) at 37 °C in a humidified air atmosphere containing 5% CO_2_.

### Skin transplantation and *in vivo* drug administration

The C57BL/6 and BALB/c mice (male, 6 weeks old, weighing 20-25 g) were anesthetized with isoflurane (concentration adjusted to 3-4%) using the VetEquip RC2 system. Subsequently, a 1 cm² section of skin was removed from the back of BALB/c (H2d) mice and transplanted onto the back of C57BL/6 (H2b) mice on POD0. The skin graft on the back of the C57BL/6 mice was secured with sutures and protected with a bandage. The C57BL/6 mice (H2b) were treated with ART (17.7 mg/kg), MMF (19.9 mg/kg), the combination of ART (17.7 mg/kg) + MMF (19.9 mg/kg), or AMNPs (ART: 17.7 mg/kg; MMF: 19.9 mg/kg) via intraperitoneal injection from POD1 to POD7. During the drug treatment period, the body weight of the mice was recorded, and the analysis of the necrosis degree and survival rate of the skin grafts was conducted. During photography and recording under optical lighting, the lighting conditions caused newly grown hair captured in the images to appear with a more distinct brownish tint.

### Pharmacokinetic evaluation of AMNPs

To investigate the pharmacokinetic characteristics of ART, MMF, and AMNPs prodrugs, SD rats were randomly divided into two groups and treated as follows: intraperitoneal injection of ART (35.4 mg/kg) + MMF (39.8 mg/kg), and intraperitoneal injection of AMNPs (with an equal amount of ART and MMF as in the previous group). Fresh blood (500 µL) was collected from the orbital venous plexus into anticoagulant tubes at 0.083, 0.166, 0.25, 0.5, 1, 2, 3, 12, 24, 31, 36, and 48 h post-administration. The blood samples were left at room temperature for 1 h, then centrifuged (4400 rpm, 10 min) to collect the plasma layer, which was stored at -80 °C for later use. The concentrations of MPA and DHA in the samples were determined.

### Flow cytometry

All the corresponding markers were detected via flow cytometry and the relevant reagents used in the primary cells and animal experiments are listed in Table S1. The C57BL/6 mice received the graft and were administered a drug for 7 days; the splenic and peripheral blood mononuclear cells were separated using the Lymphocyte Separation Solution KIT (TBD). Cells were blocked with CD16/32 to prevent nonspecific staining and stained with Fixable Viability Kit. Then cells were stained with CD3, CD4, CD8, CD44, CD62L, forkhead/winged-helix protein 3 (FOXP3), CD25, IL-4, IFN-γ, CD11b, CD11c, F4/80, CD80, CD86, MHC-II, IL-6, IL-10, TNF-α, TGF-β, and CXCR-2 antibodies. For the detection of cytokines, cells were incubated with Leukocyte Activation for 8 h, and treated with cyto-Fast Fix/Perm Buffer set (BioLegend, Cat# 426803). Then, cells were detected by flow cytometry.

### Histopathological analysis and biochemical detection

The healthy C57BL/6 mice received grafts and were treated with drugs, then the grafts and vital organs were collected. Organs were fixed and stained with H&E. For immunohistochemical analysis, we first stained the sections with anti-CD4 antibody (Cat# ab183685, Abcam), anti-CD8 antibody (Cat# ab217344, Abcam), anti-F4/80 antibody (Cat# ab300421, Abcam), and CD31 antibody (Cat# ab182981, Abcam), and then stained them with HRP-anti-rabbit IgG (Cat# AS053, Abclonal). For biochemical analysis of serum, the ALT, AST, ALP, CR, BUN, and LDH in the serum were detected.

### Immunofluorescence and living image analysis

BMDMs were cultured for 5 days and treated with ATR (10 μM), MMF (10 μM), the combination of ATR (10 μM) and MMF (10 μM), or AMNPs (10 μM). Next, BMDMs were treated with 100 ng/mL LPS and 20 ng/mL IFN-γ for 24 h and stained with DAPI, anti-MHC-II antibody and anti-CD206 antibody, the fluorescent expression of BMDMs was detected via Lecia STELLARIS 5. ART-MMF Nanoparticles were labeled with DIR, C57BL/6 mice were injected with DIR labeled AMNPs via the tail vein. After 6, 12, 24, 48, and 72 h, the axillary lymph nodes and spleens were harvested for *ex vivo* imaging. The data were analysed by Living Image software.

### Bone marrow-derived macrophages polarized activation assay and co-culture with T cells

BMDMs were isolated from C57BL/6 mice, and cultured in RPMI-1640 medium and treated with drugs for 5 days. For the induction of M1 macrophages, cells were incubated with LPS (100 ng/mL) and IFN-γ (20 ng/mL) for 24 h, and the levels of IL-1β (Cat# RK00006, Abclonal) and IL-18 (Cat# RK00104, Abclonal) in the supernatant were detected by ELISA kit. For the induction of M2 macrophages, cells were incubated with supernatant of Hepa1-6 cells for 24 h. In the end, cells were collected and the polarization markers were detected by flow cytometry. For the co-culture experiment, splenic mononuclear cells were isolated from C57BL/6 mice and cultured with M1 macrophages after drug treatment in 12-well plate stimulated with CD3&CD28 plus IL-2 (30 IU/mL) for 72 h. Subsequently, the CFSE Cell Proliferation Kit, along with CD3, CD4, CD8, IL-4, and IFN-γ antibodies, was used to detect the proliferation rate and differentiation of T cells.

### BMDMs adoptive transfer experiments

BMDMs were isolated from C57BL/6 mice. Macrophages were induced to differentiate into M0, M1, and M2 macrophage types, and M1 and M2 macrophages were treated with ATR, MMF, ATR and MMF in combination, or AMNPs at 10 μM. M0, M1, and M2 macrophage (2x10^6 cells per mouse) were injected into C57BL/6 mice via the tail vein. The C57BL/6 (H2b) mice, anesthetized with isoflurane, received the white skin from BALB/c (H2d) mice. Starting from the first day following the skin grafting surgery, the survival of the skin grafts was monitored.

### T cell adoptive transfer experiments

The C57BL/6 CD45.2 mice received the white skin from BALB/c CD45.2 mice. CD3^+^ T cells were isolated from spleen of male C57BL/6 CD45.1 mice via CD3^+^ T cells isolation kit (Cat# 480031, BioLegend). CD3^+^ T cells were incubated with CFSE dye in a 37 °C incubator for 10 minutes. CD3^+^ T cells were adoptively transferred to the C57BL/6 CD45.2 C57BL/6 mice via tail vein injection. Then C57BL/6 CD45.2 mice were treated with ART (17.7 mg/kg), MMF (19.9 mg/kg), the combination of ART (17.7 mg/kg) and MMF (19.9 mg/kg), or AMNPs (ART: 17.7 mg/kg; MMF: 19.9 mg/kg) via intraperitoneal injection for 5 days. Lymph nodes and peripheral blood were isolated from the mice to assess the proliferation rate of CD45.1^+^ CD3^+^ T cells.

### Macrophage clodronate liposomes depletion assay

The C57BL/6 (H2b) mice received the white skin from BALB/c (H2d) mice, and the skin grafts were protected with bandages. Then, clodronate liposomes were used to clear the macrophages of C57BL/6 mice. And C57BL/6 mice were treated with ART (17.7 mg/kg), MMF (19.9 mg/kg), the combination of ART (17.7 mg/kg) and MMF (19.9 mg/kg), or AMNPs (ART: 17.7 mg/kg; MMF: 19.9 mg/kg) via intraperitoneal injection every day from POD1 to POD7. On POD7, peripheral blood and splenic mononuclear cells were isolated from the mice, stained with CD3, CD4, CD8, IL-4, and IFN-γ antibodies, and analyzed to assess the effect of macrophage depletion on T-cell differentiation via flow cytometer.

### Western blotting

BMDMs were treated with ATR, MMF, ATR and MMF in combination, or AMNPs at 10 μM and incubated with 100 ng/mL LPS, 20 ng/mL IFN-γ, and 2 mM ATP. Following 24 h of stimulation, cells were harvested and then lysed using RIPA buffer. Protein concentrations were determined using a BCA assay kit. Next, the cell lysates were run on SDS-PAGE and transferred to PVDF membranes. The membranes were incubated with primary antibodies overnight at 4 °C. The next day, the membranes were washed and incubated with horseradish peroxidase-conjugated secondary antibodies for 1 h. Protein expression levels were subsequently detected. The relevant reagents are listed in Table S2.

### RNA-Seq of BMDMs

BMDMs were isolated from C57BL/6 mice and cultured in 6- well plates containing RPMI-1640 medium supplemented with AMNPs (10 μM). Then, BMDMs were stimulated with LPS (200 ng/mL) and IFN-γ (20 ng/mL) for 24 h. Following stimulation, cells were sent to HuaDa Company for RNA sequencing. RNA sequencing leverages next-generation high-throughput sequencing technologies to determine the sequences of transcriptional products and perform comparative analyses. This experiment investigates the expression patterns of eukaryotic genes and provides a precise, quantitative digital expression profile. All procedures were conducted in accordance with the operational guidelines provided by HuaDa Company.

### Statistical analysis

Statistical analyses were performed using GraphPad Prism 9.5 software and the results were expressed as mean ± SD. One-way ANOVA and student's t test were performed to analyze the differences. A value of p < 0.05 was deemed statistically significant (*p < 0.05, **p < 0.01, ***p < 0.001, ****p < 0.0001, and ns = not significant, p > 0.05).

## Supplementary Material

Supplementary materials and methods, figures.

## Abbreviations

ALP: alkaline phosphatase
ALT: alanine transaminase
ASC: Apoptosis-associated speck-like protein
ART: artesunate
AST: aspartate transaminase
BMDMs: Bone marrow-derived macrophages
BUN: blood urea nitrogen
CAS-1: Caspase-1
CR: creatinine
CFSE: carboxyfluorescencein succinimidyl ester
CMC: critical micelle concentration
CXCR-2: C-X-C motif chemokine receptor type 2
DHA: Dihydroartemisinin
DSPE-PEG2000: 1,2-distearoyl-sn-glycero-3-phosphoethanolamine-N-[methoxy(polyethylene glycol)-2000]
D_H_: hydrodynamic diameters
FOXP3: forkhead/winged-helix protein 3
GSEA: Gene set enrichment analysis
H&E: Hematoxylin & eosin
NMR: ^1^H nuclear magnetic resonance
IFN-γ: interferon-gamma
Iκ-B: Inhibitor of Nuclear Factor-κB
IL-1β: interleukin 1β
IL-2: interleukin 2
IL-4: interleukin 4
IL-6: interleukin 6
IL-10: interleukin 10
IL-18: interleukin 18
LPS: Lipopolysaccharide
MHC-II: major histocompatibility complex II
MPA: mycophenolic acid
MMF: mycophenolate mofetil
NF-κB: nuclear factor kappa B
NLRP3: NOD-like receptor family pyrin domain containing 3
PDI: polydispersity index
POD: Postoperative day
ROS: reactive oxygen species
TAC: Tacrolimus
TEM: transmission electron microscopy
Th1: Type 1 CD4^+^ T cells
Th2: Type 2 CD4^+^ T cells
TGF-β: Transforming growth factor-β
TLR-4: Toll-like receptor-4
TNF-α: Tumor Necrosis Factor-α
