# Supplementary material for: Artesunate-mycophenolate Mofetil Dimer Micelles Alleviate Allogeneic Skin Graft Rejection by Inhibiting the TLR-4 Pathway in Macrophages
Source: Theranostics. 2025 Jun 12;15(14):7154–75. doi: 10.7150/thno.108173 (PMC12204082; doi:10.7150/thno.108173)
Supplement: Supplementary file 1 — Supplementary materials and methods, figures. [file thnov15p7154s1.pdf]

Supplementary materials

Supporting Materials and Methods

1. *Materials*: Artesunate (molecular formula: $C_{19}H_{28}O_8$, molecular weight: 384.42, Cat# HY-N0193) and Mycophenolate Mofetil (molecular formula: $C_{23}H_{31}NO_7$, molecular weight: 433.49, Cat# HY-B0199) were obtained from MedChemExpress (MCE). DSPE-PEG_{2k} was obtained from the RUIXIBIO Co., Ltd. (XiAn, China). 1-(3-Dimethylaminopropyl)-3-ethylcarbodiimide (EDC) (Cat# F024810) was purchased from Bailinwei Technology Co., Ltd (Beijing, China). 4-dimethylamino pyridine (DMAP) (Cat# D1450) was purchased from Tokyo Chemical Industry (TCI, Japan). Murine IL-2 (Cat# 212-12-5UG), murine IFN-γ (Cat# 315-05-100UG) and murine M-CSF (Cat# 315-02-10UG) were purchased from Peprotech. T cell Activation/Amplification Kit was obtained from Miltenyi Biotec Co., Ltd. (Cat# 130-093-627), Cell Proliferation Kit was obtained from Thermo Fisher Scientific Co., Ltd. The CellTrace™ CFSE Cell Prolifertaion Kit (Cat# C34554) was obtained from Thermo Fisher, and lymphocyte isolation kit was purchased from TBD Co., Ltd. The clodronate liposomes (Cat# F70101C-N-10) were obtained from FoumuMax.

2. *Synthetic scheme of ART-MMF prodrug*: Artesunate and mycophenolate mofetil were dissolved in anhydrous dichloromethane. EDC and DMAP were then added dropwise, and the mixture was reacted at 45 °C overnight. The reaction was monitored using thin-layer chromatography. Once the reaction neared completion, the reaction mixture was cooled and sequentially washed with 5% citric acid, saturated sodium bicarbonate, and saturated saline to isolate and purify the intermediate ART-MMF (molecular formula: $C_{42}H_{57}NO_{14}$, Molecular weight: 799.91) conjugate. The purification results were characterized by ^1H nuclear magnetic resonance spectroscopy (NMR) and mass spectrometry (MS).

3. *Characterization of nanoparticles by DLS*: The D_H, PDI, and zeta potential of the prepared nanoparticles were characterized using dynamic light scattering (DLS) measurements on a Malvern Nano-ZS90 instrument (Malvern, UK) at a temperature of 25 °C. Each sample was measured in triplicate. The in vitro release profile of AMNPs was determined using high-performance liquid chromatography. Morphological characterization of nanoparticles by TEM: The sample solutions of ART-MMF prodrug self-assembled nanoparticles at a concentration of 0.5 mg/mL were dropped onto a 300-mesh copper grid coated with carbon. After 5 min of deposition, the surface liquid was removed with filter papers. Samples were stained with 2 wt% aqueous uranyl acetate solution for 1 min of positive staining and air-dried. The morphology of the nanoparticles was characterized using TECNAL 10 (Philips) or Talos (Thermo Scientific) at an acceleration voltage of 80 kV.

4. *PCR assay of allografts*: The mice were treated with ART (17.7 mg/kg), MMF (19.9 mg/kg), the combination of ART (17.7 mg/kg) and MMF (19.9 mg/kg), or AMNPs (ART: 17.7 mg/kg; MMF: 19.9 mg/kg) for 7 days. After sacrificing the mice, the skin grafts were

removed. Total RNA was extracted using TRIzol™reagent (MACHERY-NAGEL, Germany), then transcribed into cDNA using the HiScript III All-in-one RT SuperMix Perfect for qPCR (Vazyme, Nanjing, China) following its protocol. The prepared cDNA was subjected to qPCR analysis using a Bio-Rad CFX384 Real-time PCR System (Bio-Rad, Shanghai, China). The primers used for PCR were obtained from Tsingke Biotechnology Co., Ltd. (Beijing, China). Forward primer of CXCR-2: ATGCCCTCTATTCTGCCAGAT； Reverse primer of CXCR-2: GTGCTCCGGTTGTATAAGATGAC.

5. *Detection of serum inflammatory factors*: The mice were treated with ART (17.7 mg/kg), MMF (19.9 mg/kg), the combination of ART (17.7 mg/kg) and MMF (19.9 mg/kg), or AMNPs (ART, 17.7 mg/kg; MMF, 19.9 mg/kg) for 7 days. And the levels of IL-1β (Cat# RK00006, Abclonal) and IL-18 (Cat# RK00104, Abclonal) in the serum were detected using an ELISA kit at 7 days post-administration. The ABplex Mouse Cytokine Assay Kit (Cat# RK05203, Abclonal) was used to detect the levels of serum inflammatory factors at 28 days post-administration.

6. Detection of drug concentration in major organs: C57BL/6 mice that had undergone skin transplantation were treated with the combination of ART (17.7 mg/kg) + MMF (19.9 mg/kg), or ART-MMF nanoparticles (AMNPs: ART: 17.7 mg/kg; MMF: 19.9 mg/kg) via intraperitoneal injection, the drug concentrations of mycophenolic acid (MPA) and dihydroartemisinin (DHA) in hearts, livers, spleens, lungs, kidneys, and skin allografts were detected by mass spectrometry at 0.5 and 2 h post- administration.

Table S1

Marker	Fluorescence	Cat	Supplier
CD3	FITC	#561798	BD Biosciences
CD3	BV605	#100237	BioLegend
CD4	AF-700	#100536	BioLegend
CD4	APC	#100516	BioLegend
CD4	FITC	#100510	BioLegend
CD8	APC-cy7	#557654	BD Biosciences
CD8	PE-cy7	#100722	BioLegend
CD8	FITC	#140403	BioLegend
B220	FITC	#103205	BioLegend
CD25	BV650	#102038	BioLegend
FOXP3	PE	#126404	BioLegend
CD44	PE-CF594	#562464	BD Biosciences
CD62L	BV605	#104438	BioLegend
IL-4	PE	#504104	BioLegend
IFN-γ	BV650	#505832	BioLegend
IL-6	AF-488	#561363	BD Bioscience
IL-10	BV605	#564082	BD Bioscience
CD45.1	PE	#110707	BioLegend
CD45.2	Percp-cy5.5	#109828	BioLegend
F4/80	BV421	#123137	BioLegend
F4/80	FITC	#123108	BioLegend
F4/80	PE	#565410	BD Bioscience
CD11b	Percp-cy5.5	#101228	BioLegend
CD11b	BUV395	#563553	BD Bioscience
CD206	PE	#141706	BioLegend
MHC-II	R718	#752163	BD Bioscience
TGF-β	Percp-cy5.5	#141410	BioLegend
CD80	BV650	#104732	BioLegend
CD86	BV480	#746778	BD Bioscience
TNF-α	BV785	#506341	BioLegend
CXCR-2	BV421	#566622	BD Bioscience
CD11c	FITC	#557396	BD Bioscience
Zombie	FVS510	#564996	BD Bioscience

Table S1: Relevant antibody information used in flow cytometry.

Table S2

Antibody	Cat	Supplier
NLRP3 Rabbit mAb	#A24294	Abclonal
Nf-kb p65 Rabbit mAb	#8242s	CST
Ik-β Rabbit mAb	#A19714	Abclonal
ASC Rabbit mAb	#A22046	Abclonal
CAS Rabbit mAb	#A20470	Abclonal
IL-1 Rabbit mAb	#12242T	CST
IL-18 Rabbit mAb	#57058S	CST
TLR-4 Rabbit mAb	#14358S	CST
β-actin Rabbit mAb	#AC026	Abclonal

Table S2: Relevant antibody information used in the Western blot.

Table S3

PK parameter	MPA (ART+MMF)	MPA (AMNP)	DHA (ART+MMF)	DHA (AMNP)
$T_{1/2}$ (h)	2.981±0.8	4.258±0.792	0.2±0.0254	0.2887±0.0104
C_{max} (ug/mL)	48.23±3.2	52.6±2.607	9.65±0.804	28.63±1.250
$AUC_{(0-t)}$ (ug*h/mL)	28.431±0.572	83.42±13.61	5.22±3.047	31.04±1.326
$AUC_{(0-t)/Dose}$ (ug*kg*h/mL/mg)	0.714±0.0144	2.096±0.342	0.147±0.0086	0.876±0.0375
$AUC_{(0-\alpha)}$ (ug*h/mL)	28.434±0.573	83.46±13.6	5.230±0.3017	31.1±1.342
CL (L/h/kg)	1.40±0.283	0.4859±0.833	6.782±0.389	1.139±0.049

Table S3: Relevant parameters of pharmacokinetics, the table shows the main pharmacokinetic parameters of the relevant drug. $T_{1/2}$ represents the half-life, C_{max} indicates the peak plasma concentration, $AUC_{(0-t)}$ is the area under the plasma concentration-time curve from 0 to t , $AUC_{(0-t)/Dose}$ is the ratio of the area under the plasma concentration-time curve from 0 to t to the dose , $AUC_{(0-\alpha)}$ represents the area under the plasma concentration-time curve from 0 to infinity , and CL is the clearance rate .

Figure S1

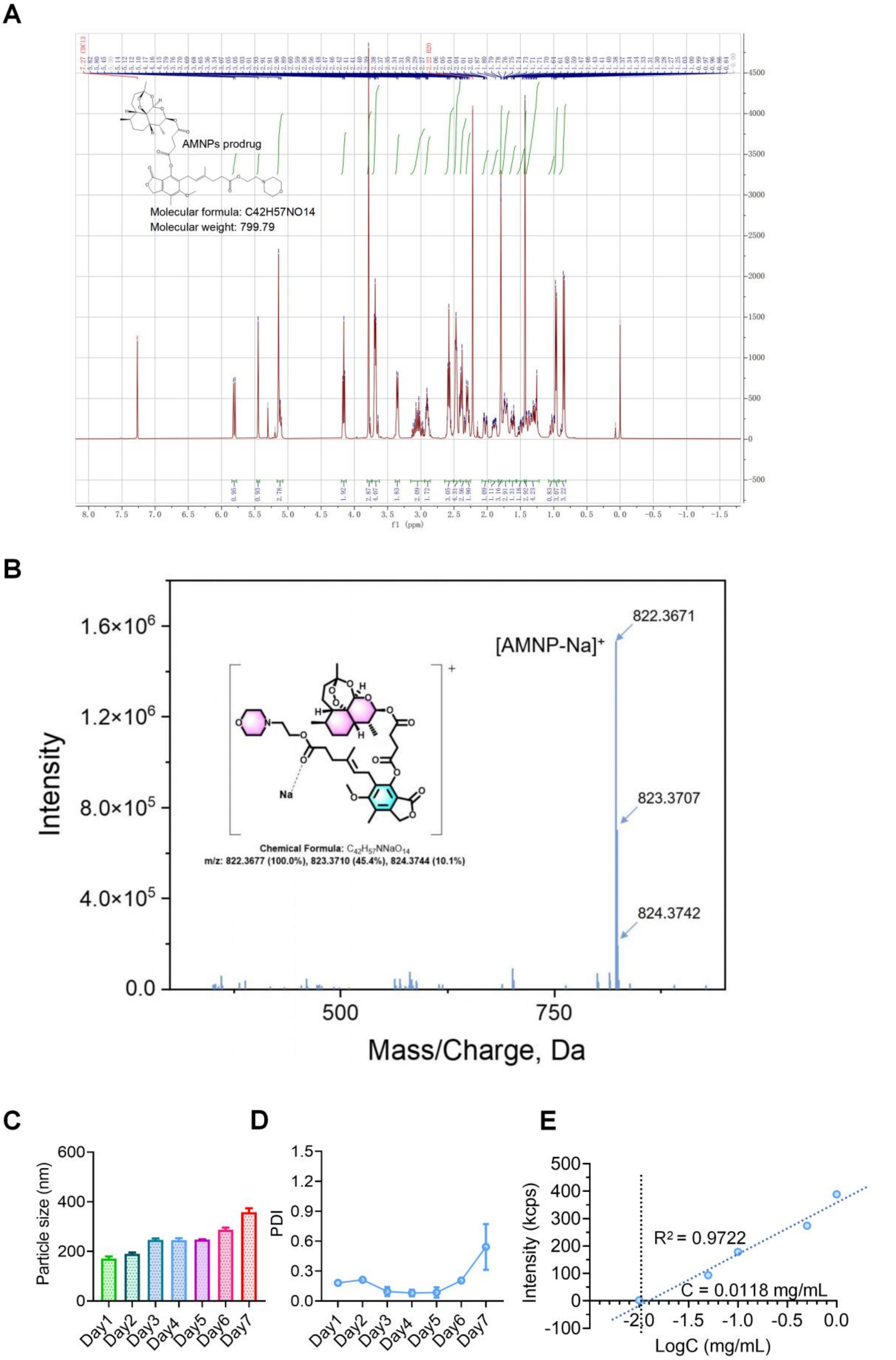

Figure S1: (A) ^{1}H NMR spectrum of AMNPs prodrug. (B) Mass spectrometry characterization of AMNPs prodrug. (C-D) Representative distribution of hydrodynamic diameter and polydispersity index of AMNPs in 5% serum (n = 3). (E) Determination for the critical micelle concentration (CMC) of AMNPs by measuring the intensity of scattered

light (kcps), the data of each group are statistically analyzed through the mean value (n = 3).

Figure S2

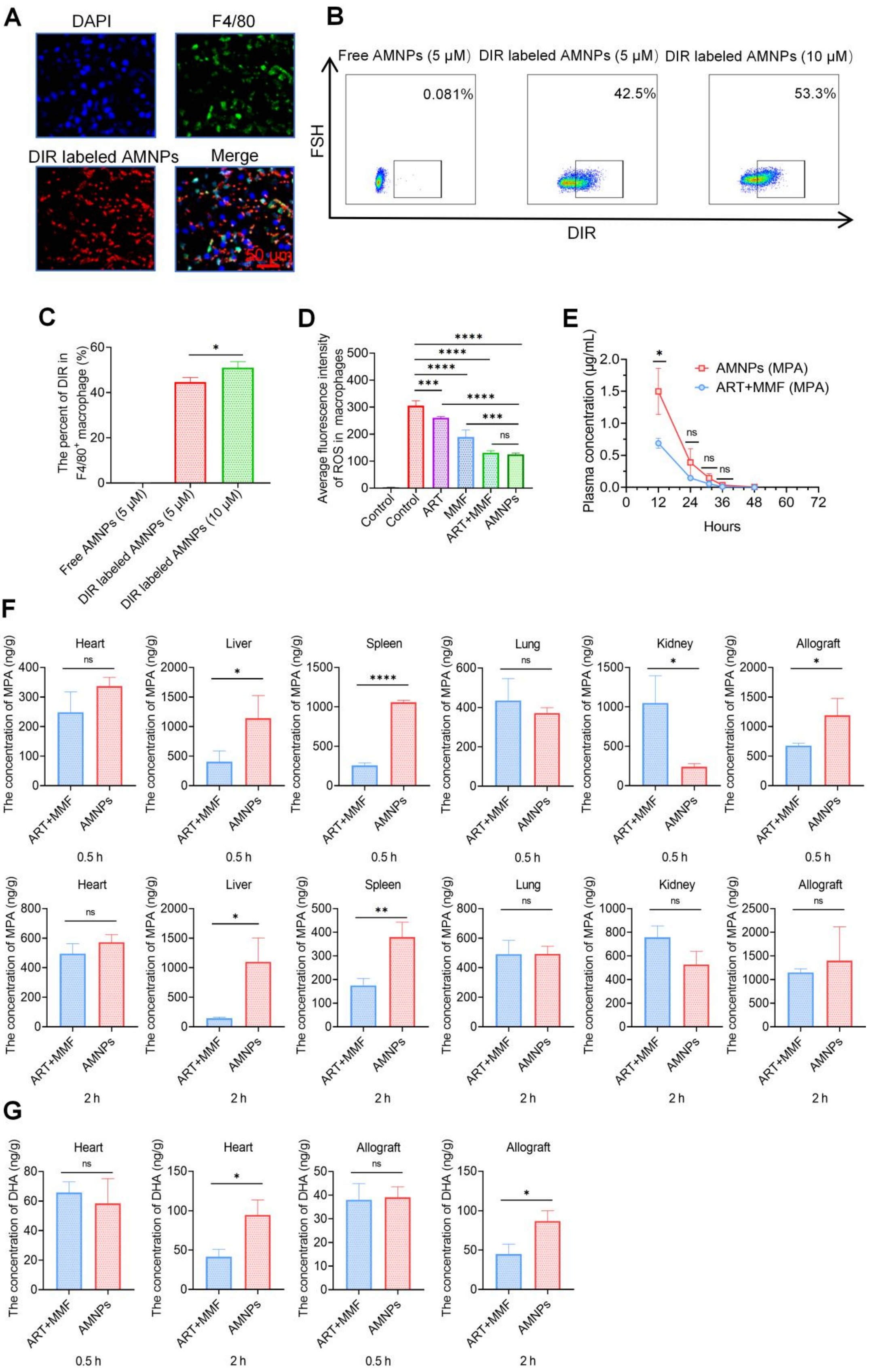

Figure S2: The accumulation of AMNPs in macrophages and their effect on ROS in

BMDM and pharmacokinetic evaluation of AMNPs. (A) Immunofluorescence showed that BMDM phagocytosed DIR labeled AMNPs. Scale bar: 50 μm (n = 3). (B) Typical flow cytometry plot showed that BMDMs phagocytosed DIR labeled AMNPs at 5 μM and 10 μM in 3 h. (C) The statistics of BMDMs phagocytosed DIR labeled AMNPs (n = 3). (D) Average fluorescence intensity of ROS in macrophages treated with ART, MMF, the combination of ART + MMF, and AMNPs, with or without LPS, reactive oxygen species were detected by using the fluorescent probe DCFH-DA (n = 6). (E) The pharmacokinetic evaluation of AMNPs and the combination of ART and MMF in SD rats was conducted at 12, 24, 31, 36, and 48 h post-administration (n = 3). (F, G) The C57BL/6 mice accepted skin transplantation were treated with the combination of ART (17.7 mg/kg) + MMF (19.9 mg/kg), or AMNPs (ART：17.7 mg/kg; MMF：19.9 mg/kg) via intraperitoneal injection, the drug concentrations of MPA and DHA in each major organ were detected by mass spectrometry at 0.5 and 2 h post- administration (n = 3). Data are represented as mean ± SD, *p < 0.05, **p < 0.01, ***p < 0.001, ****p < 0.0001, and ns = not significant, p > 0.05.

Figure S3.

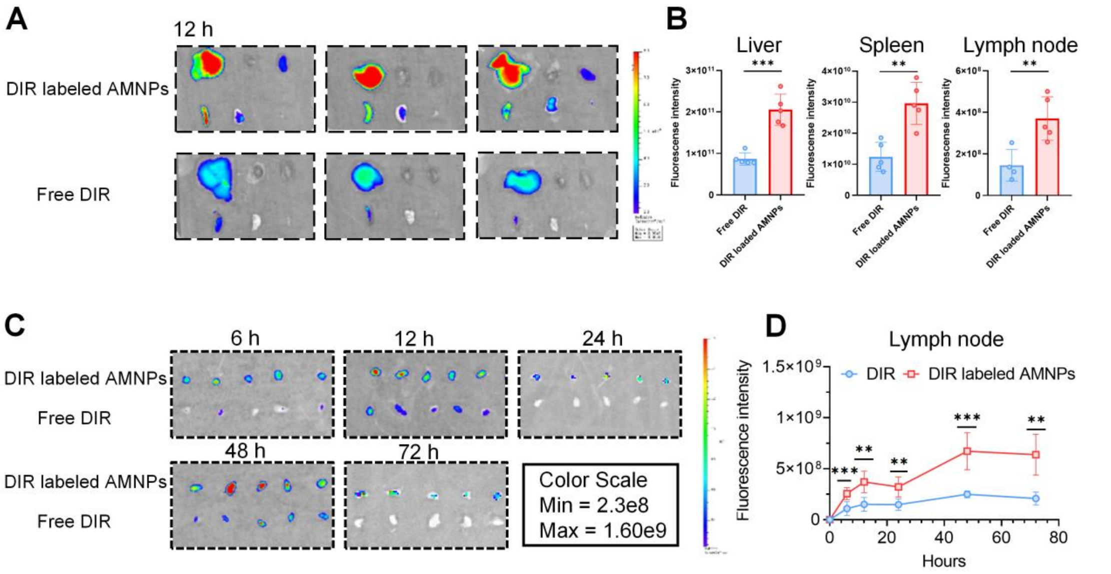

Figure S3: *In vivo* distribution of AMNPs in major organs. (A)The fluorescence images for the evaluation of free DIR or DIR labeled AMNPs distribution in heart, liver, kidney, lung, spleen, and lymph node at 12 h. (B) Quantitative analysis of DIR or DIR-labeled AMNPs accumulation in the spleen, lymph node, and liver (n = 5). (C)The fluorescence images for the evaluation of free DIR or DIR labeled AMNPs distribution in lymph node in 6, 12, 24, 48, and 72 h (n = 5). (D) Quantitative analysis of DIR or DIR labeled AMNPs accumulation in the spleen (n = 5). Data are represented as mean ± SD, *p < 0.05, **p < 0.01, ***p < 0.001, ****p < 0.0001, and ns = not significant, p > 0.05.

Figure S4

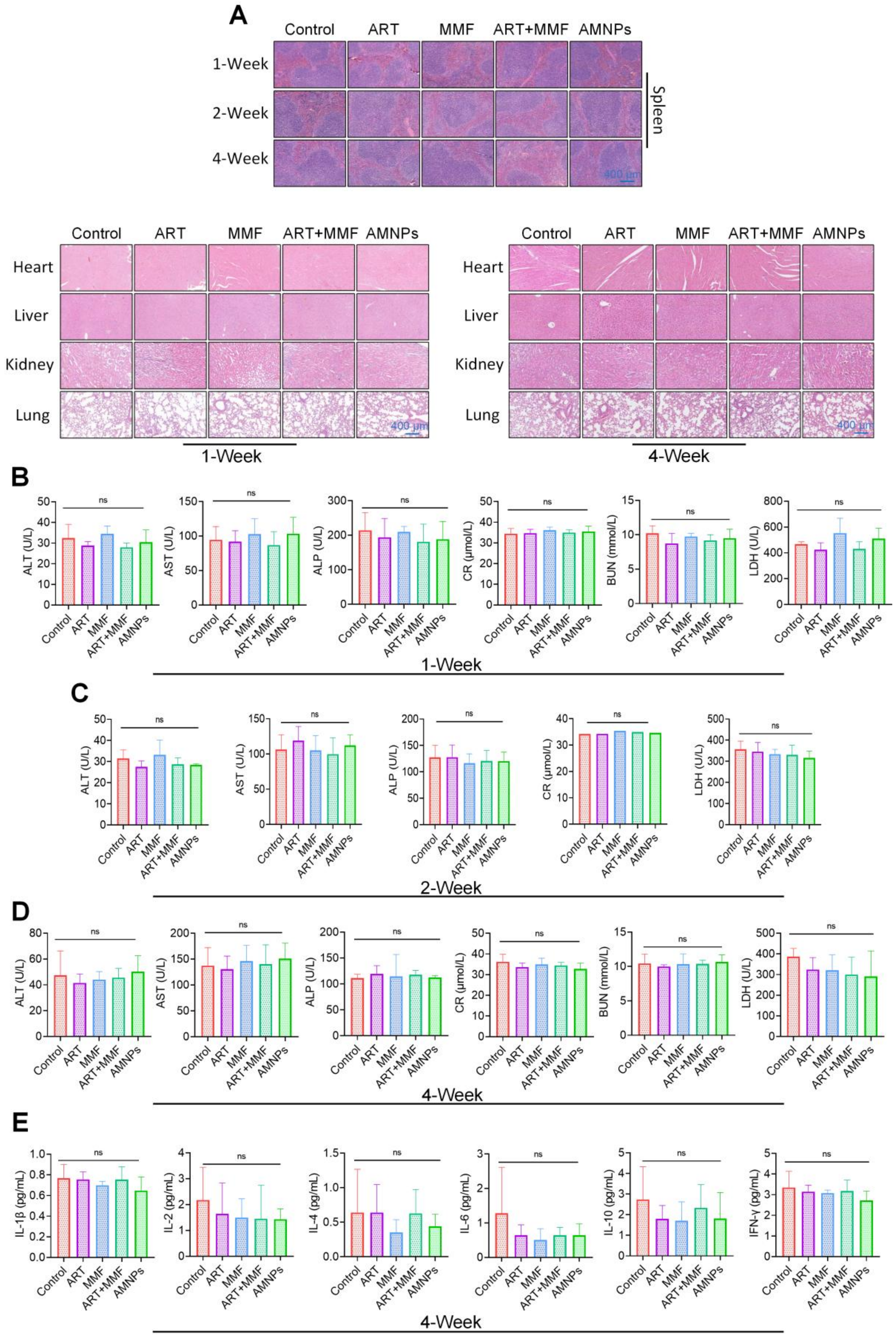

Figure S4: The effects of ART (17.7 mg/kg), MMF (19.9 mg/kg), ART (17.7 mg/kg) + MMF (19.9 mg/kg) in combination, and AMNPs (ART: 17.7 mg/kg; MMF: 19.9 mg/kg) treatment on liver and kidney toxicity in C57BL/6 mice were evaluated. (A) Representative H&E staining images of major organs from mice at 7, 14, 28 days post-administration showed

no toxic effects of the drug on these major organs. Scale bar: 400 μm (n = 5). (B) Concentrations of ALT, AST, ALP, CR, BUN, and LDH in plasma at 7 days post-administration (n = 5). (C) Concentrations of ALT, AST, ALP, CR, and LDH in plasma at 14 days post-administration (n = 5). (D) Concentrations of ALT, AST, ALP, CR, and LDH in plasma at 28 days post-administration (n = 5). (E) Concentrations of IL-1β, IL-2, IL-4, IL-6, IL-10, and IFN-γ in plasma at 28 days post-administration (n = 5). Data are represented as mean ± SD, *p < 0.05, **p < 0.01, ***p < 0.001, ****p < 0.0001, and ns = not significant, p > 0.05.

Figure S5

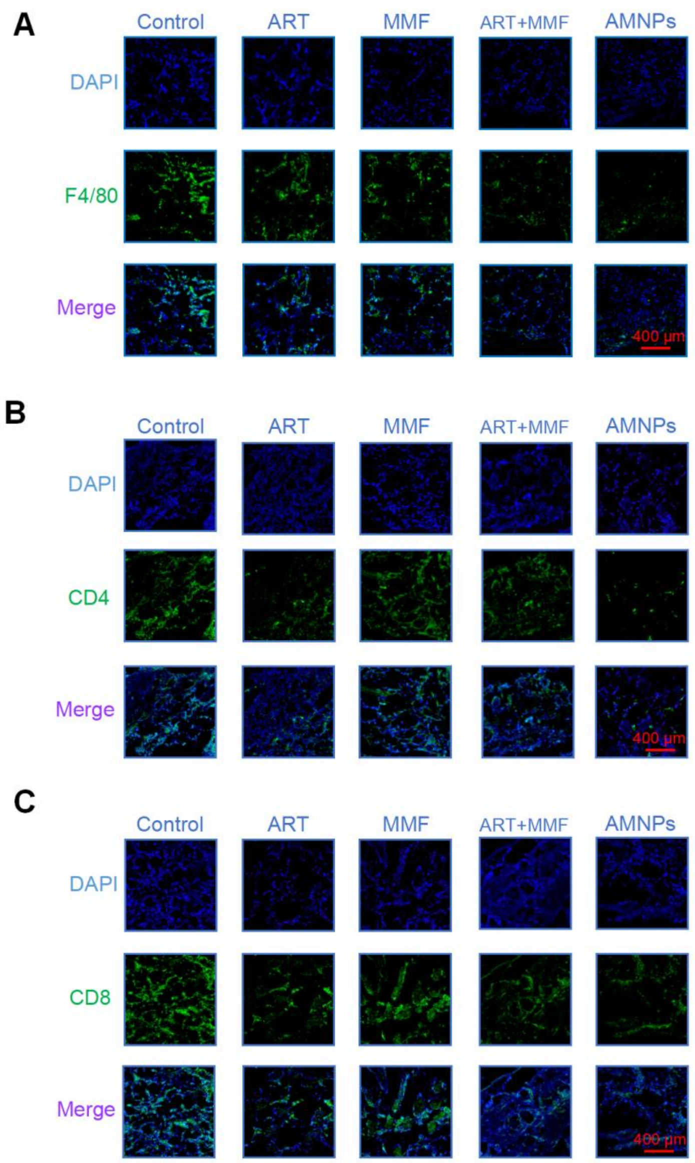

Figure S5: The effects of ART (17.7mg/kg), MMF (19.9 mg/kg), the combination of ART (17.7 mg/kg) + MMF (19.9 mg/kg) , and AMNPs (ART: 17.7 mg/kg; MMF: 19.9 mg/kg) treatment on the allogeneic skin transplantation model on POD7. (A) The immunofluorescenc of skin grafts shows the infiltration of F4/80$^+$ cells in different treatment groups. Scale bar: 400 μm (n = 3). (B) The immune fluorescence of skin grafts

shows the infiltration of $CD4^+$ cells in different treatment groups. Scale bar: 400 μm (n = 3). (C) The immune fluorescence of skin grafts shows the infiltration of $CD8^+$ cells in different treatment groups. Scale bar: 400 μm (n = 3).

Figure S6

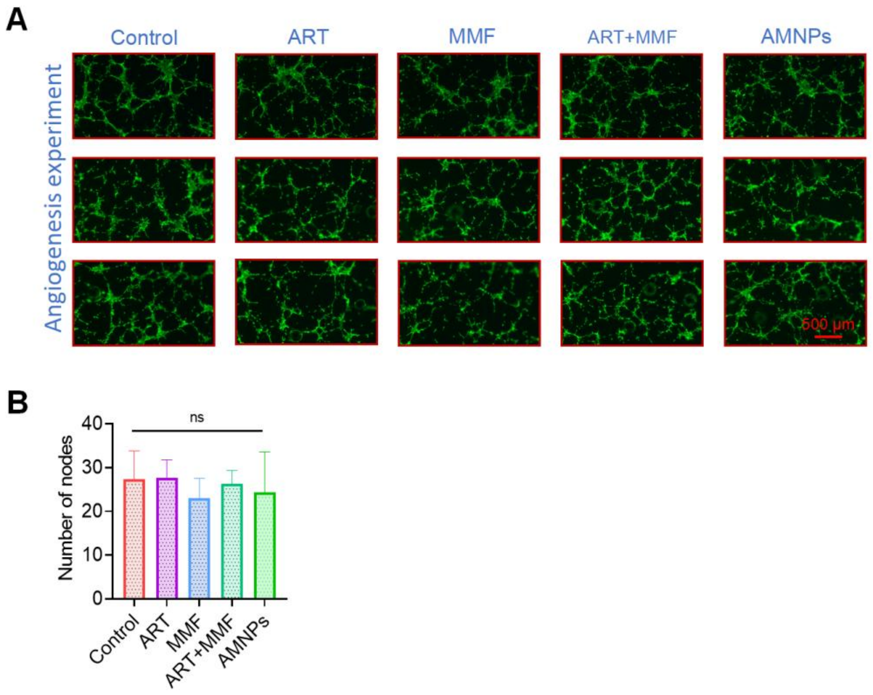

Figure S6: The effect of AMNPs on angiogenesis in HUVECs. (A) In the vascular formation experiment of HUVECs treated with ART (10 μM), MMF (10 μM), the combination of ART and MMF (10 μM), and AMNPs (10 μM), the cells were stained with calcein AM and observed under a fluorescence microscope. Scale bar: 500 μm (n = 3). (B) Quantitative statistics of vascular nodes (n=3). Data are represented as mean ± SD, *$p < 0.05$, **$p < 0.01$, ***$p < 0.001$, ****$p < 0.0001$, and ns = not significant, $p > 0.05$.

Figure S7

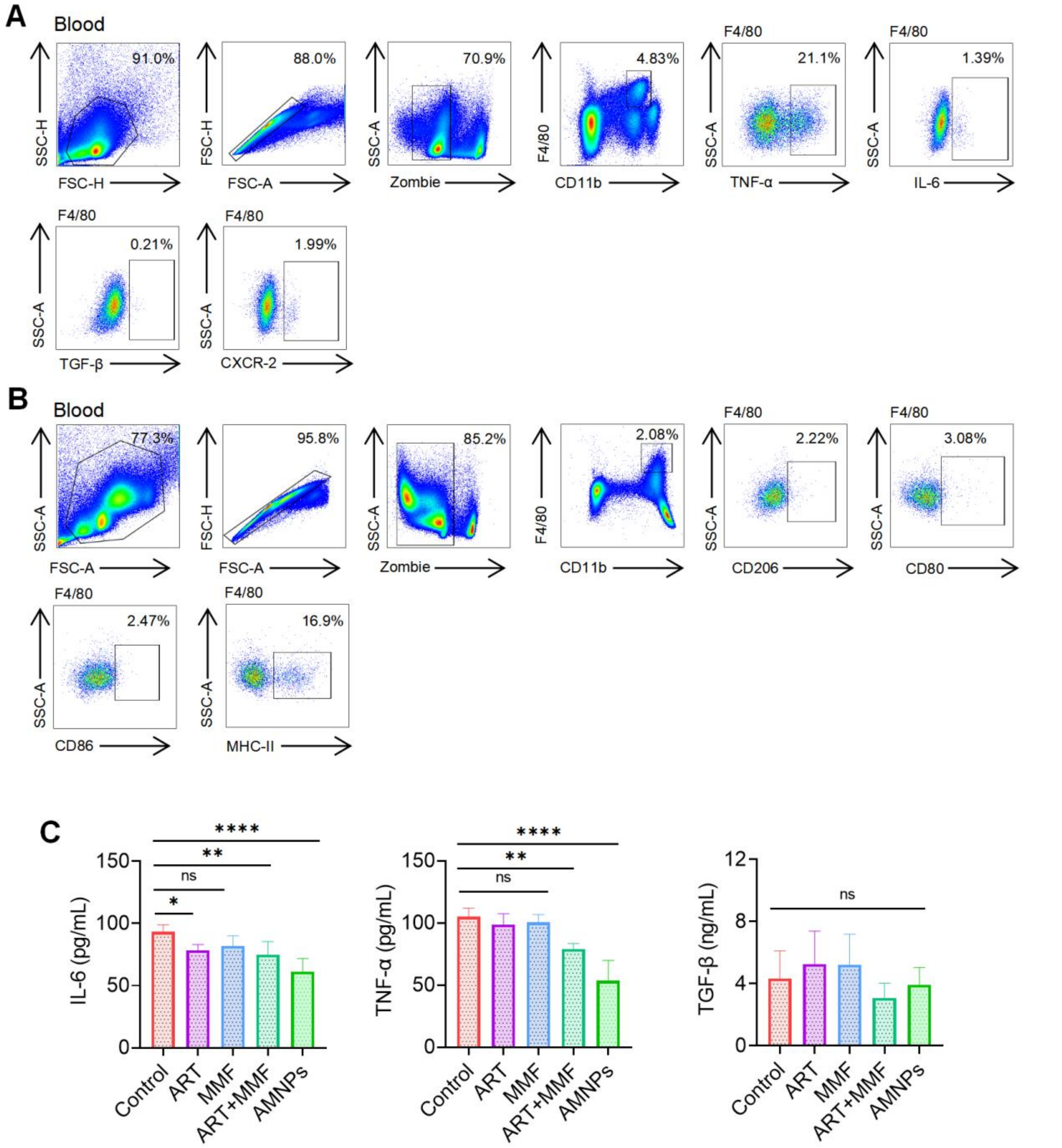

Figure S7: C57BL/6 mice received BALB/c skin grafts and were treated with ART (17.7 mg/kg), MMF (19.9 mg/kg), the combination of ART (17.7 mg/kg) and MMF (19.9 mg/kg), and AMNPs (ART: 17.7 mg/kg; MMF: 19.9 mg/kg) for 7 days. On POD7, peripheral blood and spleen mononuclear cells of C57BL/6 mice were isolated for flow cytometry analysis. On POD7, peripheral blood and spleen mononuclear cells of C57BL/6 mice were isolated and analyzed by flow cytometry. (A) Flow cytometry gating strategy diagram for pro-inflammatory and anti-inflammatory cytokines in blood macrophages. (B) Flow cytometry gating strategy diagram for blood macrophage polarization markers. (C) Concentrations of IL-6, TNF-α, and TGF-β in the serum of C57BL/6 mice were determined 7 days after drug treatment by ELISA experiment (n = 5). Data are represented as mean ± SD, *p < 0.05, **p < 0.01, ***p < 0.001, ****p < 0.0001, and ns = not significant, p > 0.05.

Figure S8

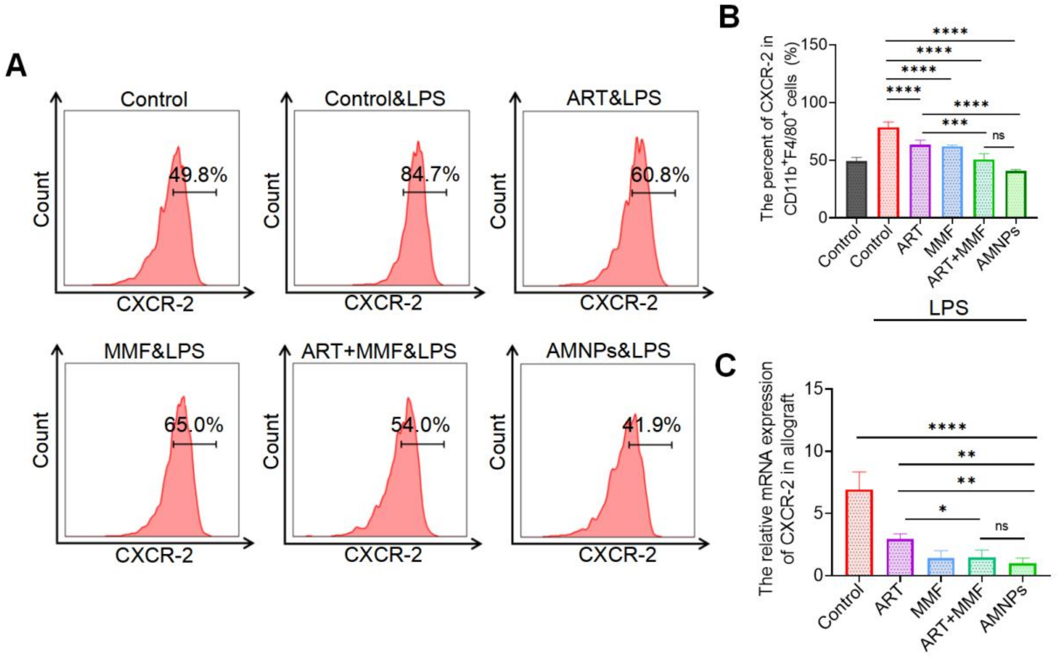

Figure S8: The effect of AMNPs on CXCR-2 expression in BMDMs and skin grafts. (A) A typical flow cytometry plot showing CXCR-2 expression in BMDMs following ART (10 μM), MMF (10 μM), the combination of ART (10 μM) and MMF (10 μM), or AMNPs (10 μM) treatment. (B) The CXCR-2 expression in F4/80^{+} macrophages (n = 4). (C) The mRNA level of CXCR-2 in allograft on POD7 (n = 4). Data are represented as mean ± SD, *p < 0.05, **p < 0.01, ***p < 0.001, ****p < 0.0001, and ns = not significant, p > 0.05.

Figure S9

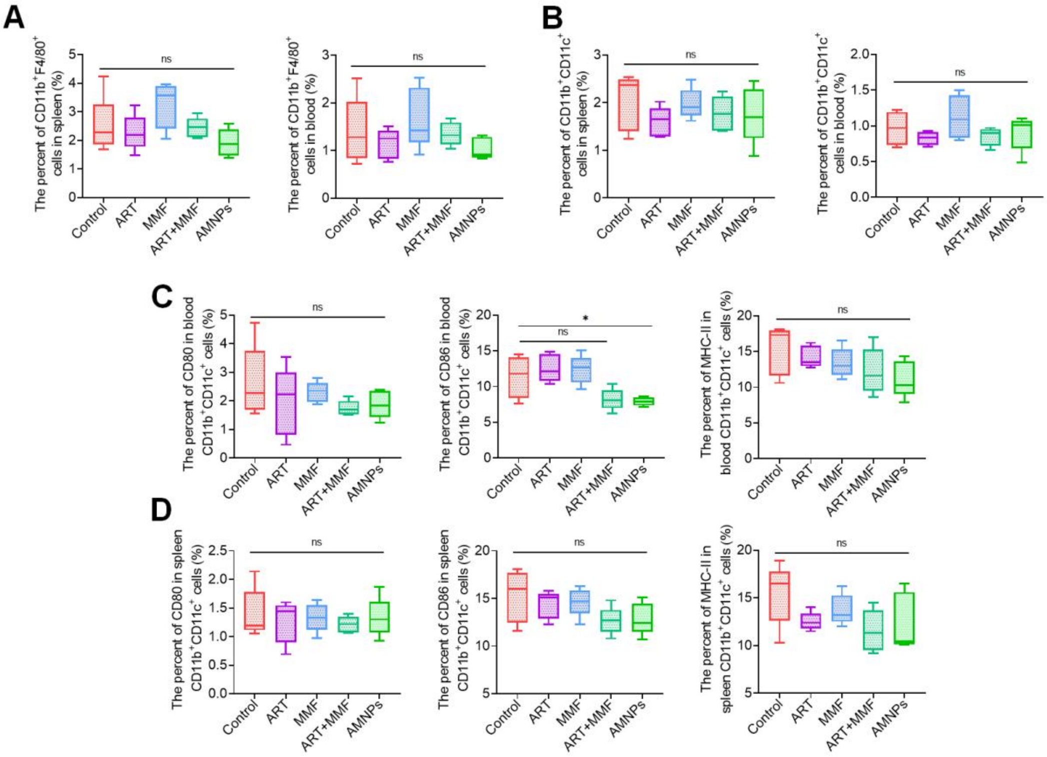

Figure S9: The effects of ART (17.7 mg/kg), MMF (19.9 mg/kg), the combination of ART (17.7 mg/kg) and MMF (19.9 mg/kg), or AMNPs (ART: 17.7 mg/kg; MMF: 19.9 mg/kg) treatment on the spleen and peripheral blood antigen-presenting cells in the allogeneic skin transplantation model after 7-day treatment. (A) Proportions of splenic and blood $CD11b^+$ $F4/80^+$ macrophages among total viable monocytes (n = 5). (B) Proportions of splenic and blood $CD11b^+CD11c^+$ cells among total viable monocytes (n=5). (C) Expression of CD80, CD86, and MHC-II expression in blood $CD11b^+$ $CD11c^+$ cells (n = 5). (D) Expression of CD80, CD86, and MHC-II expression in splenic $CD11b^+CD11c^+$ cells (n = 5). Data are represented as mean ± SD, *$p < 0.05$, **$p < 0.01$, ***$p < 0.001$, ****$p < 0.0001$, and ns = not significant, $p > 0.05$.

Figure S10

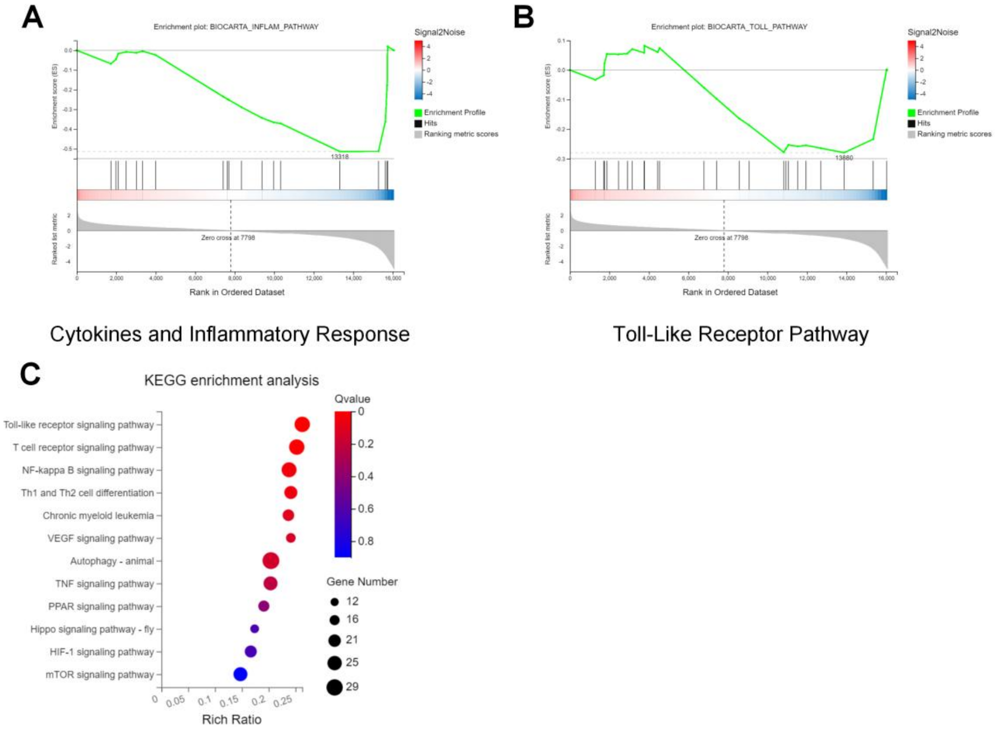

Figure S10: Transcriptome sequencing analysis was performed on BMDMs treated with PBS or AMNPs (10 μM). (A) GSEA analysis showed that the cytokines and inflammatory response in BMDMs was inhibited by AMNPs treatment (n = 6). (B) GSEA analysis showed that the Toll-Like Receptor Pathway in BMDMs was inhibited by AMNPs treatment (n = 6). (C) KEGG enrichment analysis indicated that gene expression in BMDMs was affected by AMNPs treatment (n = 6).

Figure S11

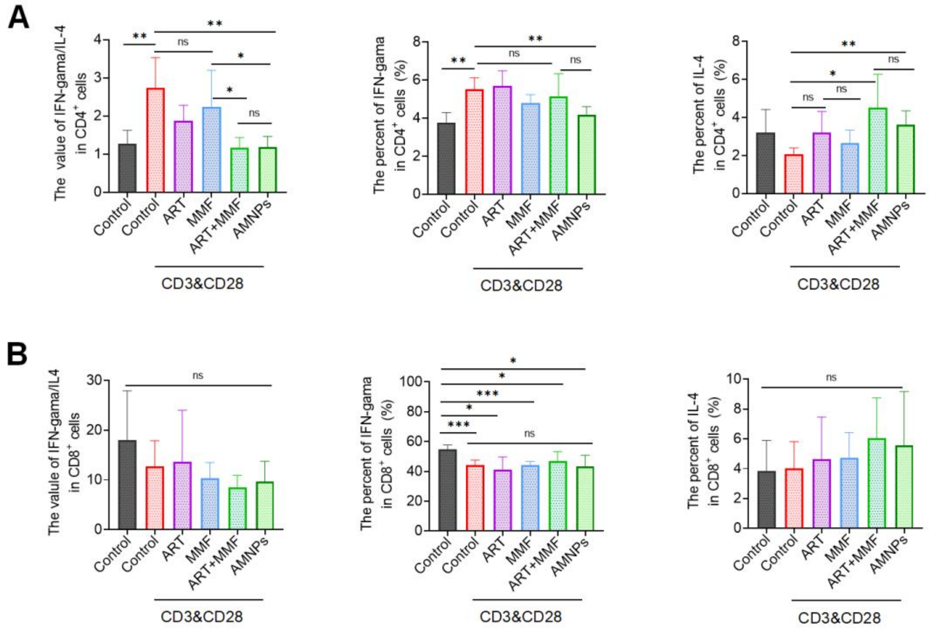

Figure S11: The effect of ART (10 μM), MMF (10 μM), the combination of ART and MMF (10 μM), and AMNPs (10 μM) treatment, with or without CD3&CD28 antibody, on the differentiation of T cells. (A) The expression of IL-4, IFN-γ, and the ratio of IFN-γ/IL-4 in $CD3^+ CD4^+$ T cells (n = 5). (B) The expression of IL-4, IFN-γ, and the ratio of IFN-γ/IL-4 in $CD3^+ CD8^+$ T cells (n = 5). Data are represented as mean ± SD, *p < 0.05, **p < 0.01, ***p < 0.001, ****p < 0.0001, and ns = not significant, p > 0.05.

Figure S12

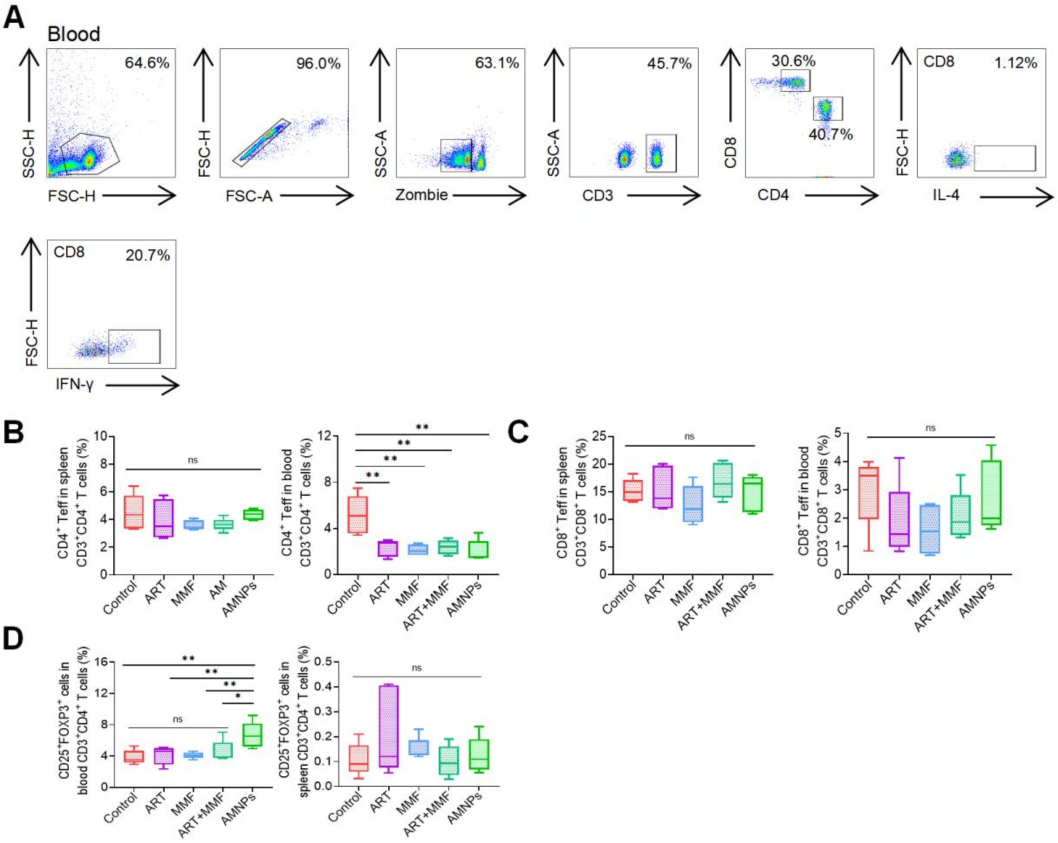

Figure S12: C57BL/6 mice received BALB/c skin grafts and were treated with ART (17.7 mg/kg), MMF (19.9 mg/kg), the combination of ART (17.7 mg/kg) and MMF (19.9 mg/kg), and AMNPs (ART: 17.7 mg/kg; MMF: 19.9 mg/kg) for 7 days. On POD7, peripheral blood and spleen mononuclear cells of C57BL/6 mice were isolated for flow cytometry analysis. (A) Flow cytometry gating strategy diagram for the differentiation of blood T cells. (B) The proportion of $CD44^{high}$ $CD62L^{low}$ T cells in splenic and blood $CD3^{+}$ $CD4^{+}$ T cells (n = 5). (C)The proportion of $CD44^{high}$ $CD62L^{low}$ T cells in splenic and blood $CD3^{+}$ $CD8^{+}$ T cells (n = 5). (D) The proportion of $CD25^{+}$ $FOXP3^{+}$ T cells in splenic and blood $CD3^{+}$ $CD4^{+}$ T cells (n = 5). Data are represented as mean ± SD, *p < 0.05, **p < 0.01, ***p < 0.001, ****p < 0.0001, and ns = not significant, p > 0.05.

Figure S13

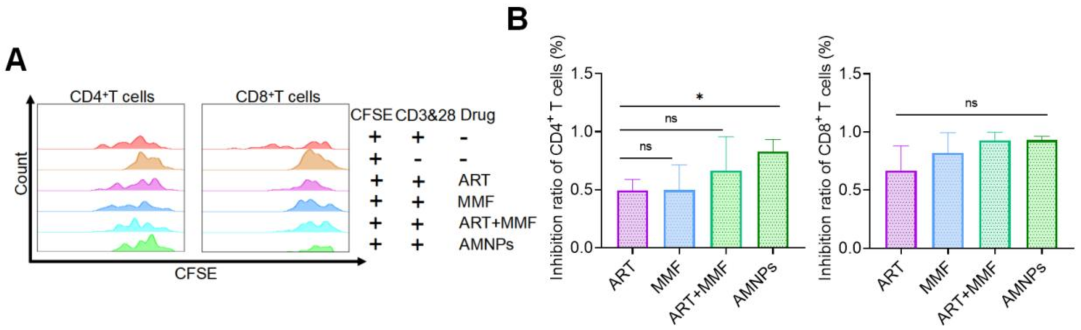

Figure S13: The effect of ART (10 μM), MMF (10 μM), the combination of ART + MMF (each at 10 μM), and AMNPs (10 μM) treatment on differentiation of T cells *in vitro*. (A) Representative flow cytometry histogram showed the inhibitory effect of ART, MMF, the combination of ART and MMF, or AMNPs on T cells at different concentrations. (B) The inhibition ration of CD4$^+$ and CD8$^+$ T cells (n = 3). Data are represented as mean ± SD, *p < 0.05, **p < 0.01, ***p < 0.001, ****p < 0.0001, and ns = not significant, p > 0.05.

Figure S14

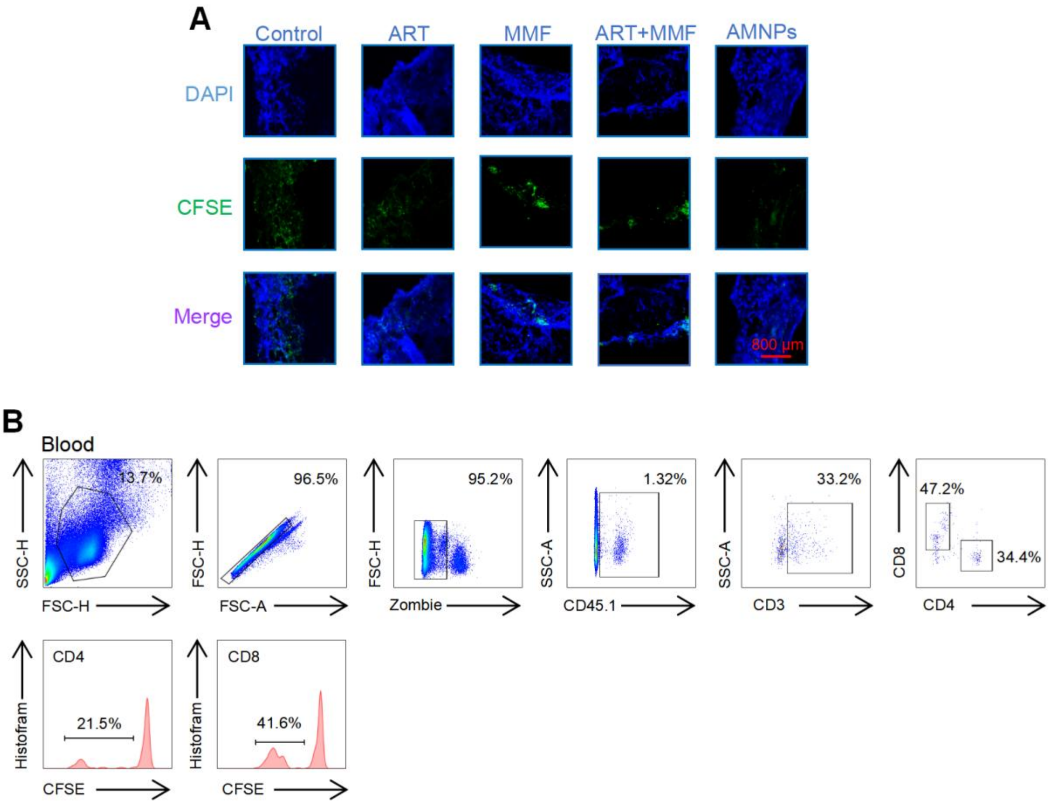

Figure S14: In allogeneic skin transplantation in CD45.2 C57BL/6 mice, CFSE labeled mononuclear cells were adoptively transferred, and treated with ART (17.7 mg/kg), MMF (19.9 mg/kg), the combination of ART (17.7 mg/kg) and MMF (19.9 mg/kg), or AMNPs (ART: 17.7 mg/kg; MMF: 19.9 mg/kg) for 5 days. (A) Immunofluorescence experiments

showed the infiltration of CFSE labeled mononuclear cells in skin grafts on POD5. Scale bar: 800 μm (n = 3). (B) Flow cytometry gating strategy diagram for CFSE adoptive transfer experiments in blood samples.
